# Improved ^140^Nd Production for the ^140^Nd/^140^Pr In Vivo Generator through Target Recycling and Radiochemical Optimization

**DOI:** 10.1021/acsomega.6c03114

**Published:** 2026-07-18

**Authors:** Mangi Lal Godara, Gabriel Dufour, Anna Buryachenko, Suzanne E. Lapi

**Affiliations:** † Department of Chemistry, 9968 University of Alabama at Birmingham, Birmingham, Alabama 35294, United States; ‡ Department of Radiology, University of Alabama at Birmingham, Birmingham, Alabama 35294, United States; § Department of Chemical and Materials Engineering, 7161 San Jose State University, San Jose, California 95192, United States

## Abstract

Theranostic strategies that utilize f-block therapeutic radionuclides, including ^161^Tb, ^177^Lu, ^225^Ac, and ^227^Th, suffer from a shortage of positron emission tomography (PET) imaging counterparts in the same chemical space and often rely on ^68^Ga as a surrogate. The ^140^Nd/^140^Pr in vivo PET generator, which belongs to the f-block, may address this issue and can be produced via the ^141^Pr­(p,2n)^140^Nd production route by using medium-energy cyclotrons. However, impurities in the target material, including stable Nd, and the inherent difficulty of adjacent lanthanide separations limit the achievable radionuclidic and chemical purity of ^140^Nd. In this work, we address these challenges through the purification and recycling of praseodymium target material and optimization of Nd/Pr separation. The resulting purified ^140^Nd was evaluated using DOTA and Macropa chelators via radiolabeling and in vitro stability studies. A target material purification and recycling method was developed for the monoisotopic ^141^Pr starting material to remove stable Nd impurities, yielding 90.3 ± 4.7% (*n* = 3) recovery. The purified ^141^Pr was isolated as Pr_6_O_11_ and irradiated with 24 MeV protons (20.07 MeV at the target surface) at 20 μA for 4 h, which produced 1417.0 ± 83.4 MBq (38.3 ± 2.2 mCi) of ^140^Nd at the end of bombardment (EOB). The produced ^140^Nd was purified through an optimized DGA normal method to recover 71.6 ± 6.3% pure ^140^Nd. The amount of stable Nd reduced progressively in each target purification cycle from >340 ppm without purification to <250 ppb after three cycles, while other measured metallic impurities were below 30 ppb. This improvement in target purity was reflected in the direct increase of apparent molar activity (AMA), when purified ^140^Nd was evaluated with DOTA and Macropa chelators. AMA of [^140^Nd]­Nd-DOTA and [^140^Nd]­Nd-Macropa increased from 70.3 MBq/μmol (1.9 mCi/μmol) and 74 MBq/μmol (2.0 mCi/μmol) to 8025.3 MBq/μmol (216.9 mCi/μmol) and 8473.0 MBq/μmol (229.0 mCi/μmol), respectively, after the third target purification cycle. Further evaluation of chelator-labeled ^140^Nd showed that [^140^Nd]­Nd-DOTA was stable in phosphate-buffered saline (PBS), saline, human serum, and mouse serum, whereas [^140^Nd]­Nd-Macropa was stable in all except human serum. This work established a practical methodological advance for the production of ^140^Nd/^140^Pr in vivo PET generators, combining optimized target recycling and radiochemical separation to enable scaled-up and high-molar activity ^140^Nd suitable for preclinical imaging. These advances support broader development of ^140^Nd/^140^Pr as a robust PET analogue, especially for f-block therapeutics.

## Graphical abstract

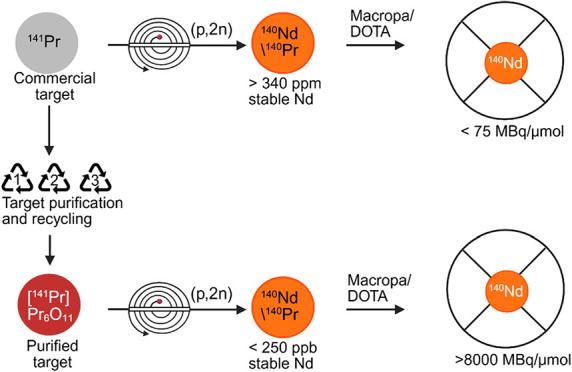

## Introduction

Theranostic strategies which integrate diagnostic imaging using positron emission tomography (PET) or single-photon emission computed tomography (SPECT) with targeted radionuclide therapy (TRT) can be used to improve treatment outcomes through patient selection, dosimetry, and response prediction.^1–3^ F-block therapeutic radionuclides, including ^161^Tb (*t*_1/2_: 6.9 d), ^177^Lu (*t*_1/2_: 6.7 d), ^225^Ac (*t*_1/2_: 9.9 d), and ^227^Th (*t*_1/2_: 18.7 d), have contributed significantly to the recent success of therapeutic radiopharmaceuticals. The FDA-approved agents including [^177^Lu]­Lu-DOTATATE and [^177^Lu]­Lu-PSMA-617 and a growing number of ongoing clinical trials, including [^225^Ac]­Ac-PSMA-617, [^225^Ac]­Ac-PSMA-I&T, [^225^Ac]­Ac-J591, [^225^Ac]­Ac-lintuzumab, and [^161^Tb]­Tb-PSMA I&T, highlight the impact of this strategy.^4–7^ However, because of a lack of PET-compatible surrogates for these f-block therapeutic radionuclides, the commonly used PET radionuclide ^68^Ga (*t*_1/2_: 67.7 min, 88.9% β^+^) is often utilized to predict tumor uptake and assess treatment response for therapies such as [^177^Lu]­Lu-DOTATATE and [^177^Lu]­Lu-PSMA-617.^8,9^ However, Ga belongs to the p-block and may reflect different coordination chemistry. The chemical behavior of ^68^Ga combined with the short half-life can hinder the use of this radionuclide for certain applications. To address this critical gap of PET-compatible diagnostic counterparts for f-block therapeutic radionuclides, the in vivo PET generator system neodymium-140 (^140^Nd (*t*_1/2_: 3.4 d, EC))/praseodymium-140­(^140^Pr (*t*_1/2_: 3.4 min, 51.0% β^+^)) offers a promising strategy.^10–12^ In vivo PET generator systems rely on the decay of a long-lived parent radionuclide to generate a short-lived positron-emitting daughter in vivo, enabling extended PET imaging without repeated tracer administration. Due to its chemical similarity to other f-block elements, the ^140^Nd/^140^Pr generator system is expected to exhibit pharmacokinetics closely resembling those of therapeutic lanthanides and actinides, making it an attractive PET imaging surrogate.

There are several potential production routes for ^140^Nd including ^140^Ce­(^3^He,3n)^140^Nd; ^140^Ce­(α,4n)^140^Nd; ^142^Ce­(^3^He,5n)^140^Nd; ^142^Ce­(α,6n)^140^Nd; ^141^Pr­(p,2n)^140^Nd; ^141^Pr­(d,3n)^140^Nd; ^141^Pr­(t,4n)^140^Nd; ^141^Pr­(^3^He,x)^140^Nd; and ^141^Pr­(α,x)^140^Nd.^13–18^ The ^141^Pr­(p,2n)^140^Nd reaction route has a high cross section with a peak window between 18 and 24 MeV proton energies. The cross-section and production of ^140^Nd have been explored by Hogan, Hilgers et al., Zeisler et al., Becker et al., and Aikawa et al. via the same reaction.^13,14,19–22^ The cross-section determinations by Aikawa et al. indicates the peak at 22.2 MeV proton energy with a cross-section value of 956 ± 85 mb.^13^ This 18−24 MeV energy range is suitable for medium-energy cyclotrons to produce yields of ^140^Nd to sustain preclinical research and may potentially support clinical applications. Only a few reports, including Zhernosekov et al., Rösch et al., and Godara et al., have investigated the production and purification of ^140^Nd and suggest that the radiochemical purity remains a challenge in processed ^140^Nd, limiting its use in preclinical applications.^10,11,23^ In our previous work, ^140^Nd production via Pr foil and development of a separation method established a robust route for acquiring ^140^Nd; however, the highest-purity ^141^Pr metal obtained via commercial sources contained significant impurities, including 0.050 ± 0.001% cerium (Ce), 1.050 ± 0.037% neodymium (Nd), and 1.070 ± 0.028% gadolinium (Gd), as analyzed by ICP-MS; thus, stable Nd impurities originating from the ^141^Pr target material resulted in essentially carrier-added ^140^Nd, limiting attainable molar activity and restricting preclinical applicability.^10^ Additionally, the recovery yield of the ^140^Nd from the purification could be improved with prior reported yields of ∼27%. The adjacent lanthanides Nd/Pr are specifically challenging to separate owing to their similar chemical properties, including the only stable +3 oxidation state, unlike Ce, which can achieve a +4 oxidation state, thus creating a chemical difference which can be utilized for separation.^11^ To establish the ^140^Nd/^140^Pr in vivo PET generator as a robust PET imaging surrogate for f-block therapeutic radionuclides, these key production and purification barriers must be addressed. The aim of this study was to convert the previously feasible but impurity and scale-up-limited production route into a practical method for obtaining high-purity ^140^Nd suitable for radiolabeling and biological evaluation. Herein, we report on the development of a target recycling method for the purification of the ^141^Pr target material, an oxide target for the production of ^140^Nd, and an optimized purification method to obtain high-purity ^140^Nd with improved recovery. The purified ^140^Nd was subsequently evaluated through radiolabeling of DOTA and Macropa chelators, followed by in vitro studies including the stability of labeled chelators in physiological conditions and free ^140^Nd binding in blood to assess its suitability for translational applications.

## Results

### Target Purification and Recycling

The commercially sourced monoisotopic ^141^Pr metal foil contained stable Ce, Nd, and Gd as major impurities among others (Table S1). A ^141^Pr target purification and recycling method was developed to obtain a high-purity target material. As stable Nd may end up in the final purified ^140^Nd assay as a carrier-added impurity, removal of stable Nd from the target material Pr is an important step in the overall target purification cycle. Since the adjacent lanthanides Nd and Pr have highly similar chemical properties, their separation is particularly challenging. The normal DGA resin, which contains embedded *N*,*N*,*N*′,*N*′-tetraoctyldiglycolamide groups, has a Nd/Pr separation factor of 2.5, the highest among LN, LN2, LN3, and branched DGA resins, and weight distribution constants (*D*_w_) of ∼12 for Pr and ∼30 for Nd in 3 M HCl. Hence, this resin was used to remove stable Nd from Pr for the target purification as well as for radionuclide separation downstream. One purification/recycling cycle consisted of (i) target dissolution, (ii) separation of impurity-primarily stable Nd from Pr using a DGA normal resin column, (iii) isolation of the purified target material Pr_6_O_11_, and (iv) pellet fabrication for subsequent irradiation. The initial Pr target was dissolved using con. HCl and diluted with 4 M HCl for the separation step. Pr was separated from Nd using the column with 2.7 M HCl as a washing solvent. The recovered Pr from the separation step was hydrolyzed to generate the Pr­(OH)_3_ precipitate, which was filtered, dried, and heated up to 800 °C to obtain Pr_6_O_11_. In a single recycling cycle, 90.3 ± 4.7% Pr was recovered. Thermogravimetric analysis (TGA) of Pr­(OH)_3_, performed at a heating rate of 5 °C/min up to 800 °C, showed a total weight loss of 24.8% (Figure 1). The initial gradual weight loss of around ∼10% from 30 to 280 °C can be attributed to the evaporation of the absorbed content, including moisture. A sharp weight loss was observed from 280 to 450 °C, corresponding to the conversion of Pr­(OH)_3_ to intermediate praseodymium oxides, while a very gradual loss in weight was observed above 450 °C, yielding dark-brown powder, which can be attributed to the conversion of praseodymium oxides to Pr_6_O_11_.^24^ Scanning electron microscopy (SEM) analysis of the gold-sputtered Pr_6_O_11_ pellet revealed a relatively smooth surface and uniform morphology (Figure 2a,b). The SEM-EDX (energy dispersive X-ray) elemental analysis of the pellet surface indicated 36.84% Pr, 60.02% O, and 3.14% Cl contents (Figure 2c).

**Figure 1:**
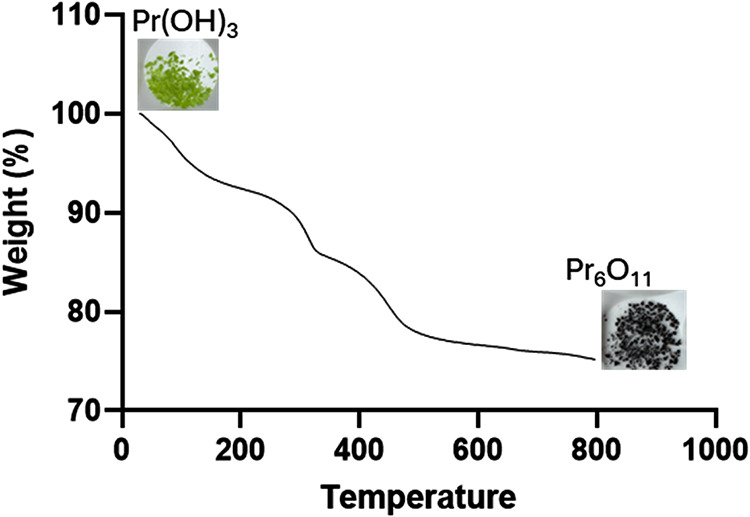
Thermogravimetric analysis (TGA) curve of Pr­(OH)_3_ powder heating at a constant increasing temperature of 5 °C/min from 30 to 800 °C.

**Figure 2:**
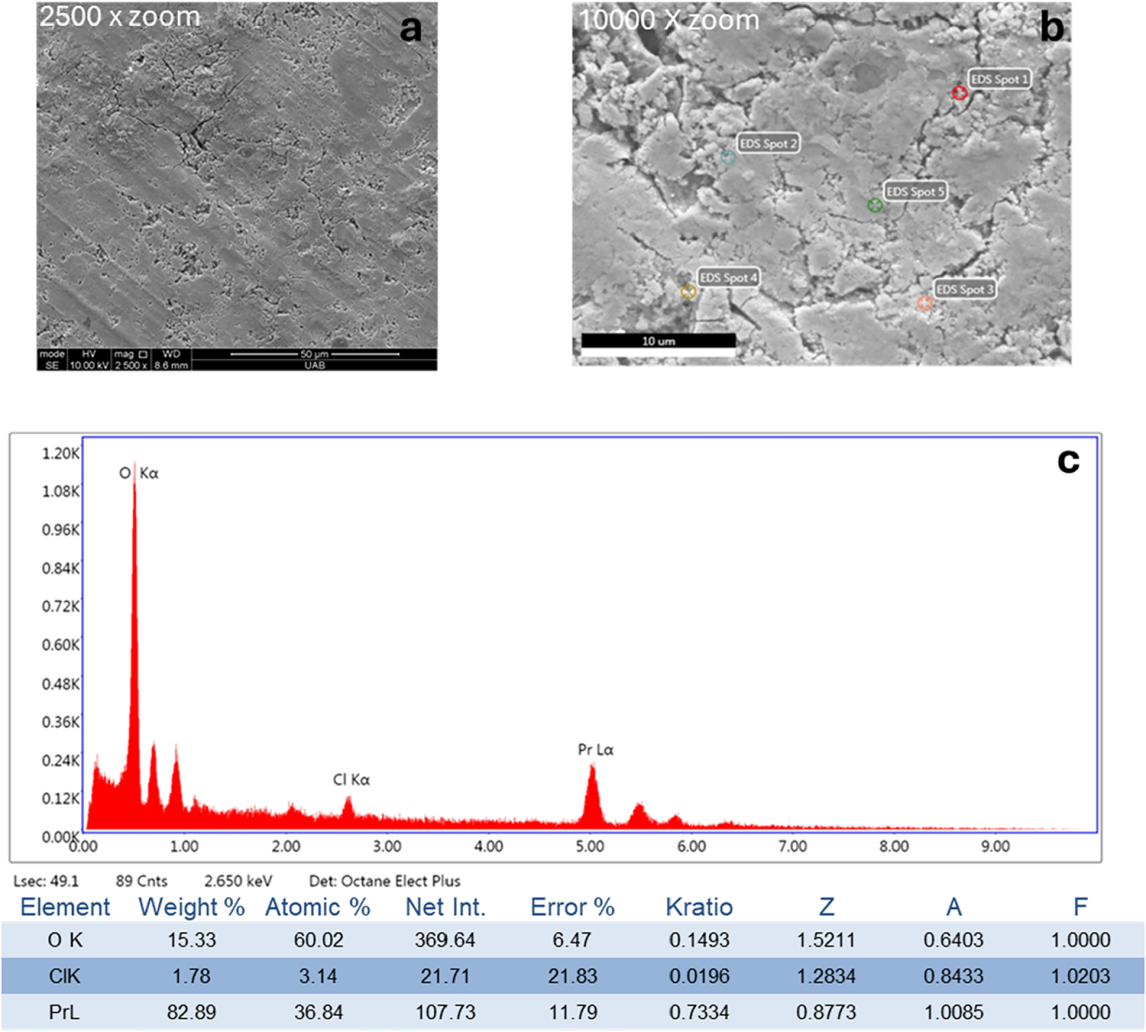
Scanning electron microscopy (SEM) analysis of the Pr_6_O_11_ pellet. (a) Pr_6_O_11_ pellet surface morphology zoomed in 2500 times to the original size. (b) Pr_6_O_11_ pellet surface morphology zoomed in 10,000 times to the original size. (c) SEM-EDX elemental analysis on the pellet surface.

### Target Irradiation

The irradiation target consisted of a 100 mg monoisotopic [^141^Pr]­Pr_6_O_11_ pellet (0.34 mm thickness, 10.21 mm diameter) fabricated by applying a stepwise increasing pressure of up to 6 t to dried powder by using a hydraulic press. The target was irradiated with a 24 MeV proton beam from the Al degrader side. The Stopping and Range of Ions in Matter (SRIM-2013) simulations showed that the beam energy decreased to 20.07 MeV at the Pr_6_O_11_ surface and further decreased to 18.18 MeV upon exiting the pellet before being completely stopped within the tantalum backing. The entrance and exit energies of the Pr_6_O_11_ pellet lie around the peak cross section of the ^141^Pr­(p,2n)^140^Nd reaction (Figure S1b). Other side products including ^141^Nd (*t*_1/2_: 2.5 h), ^139m^Nd (*t*_1/2_: 5.5 h), and ^139^Ce (*t*_1/2_: 137.6 d) can be produced via nuclear reactions ^141^Pr­(p,n)^141^Nd, ^141^Pr­(p,3n)^139m^Nd, and ^141^Pr­(p,x)^139^Ce at the irradiated proton energy window (Figure S1).

To minimize these side reactions while maintaining a high production yield of ^140^Nd, the optimized proton energy upon the target interaction needed was close to 20 MeV. Based on the Al degrader’s thickness, when the target was irradiated at 24 MeV, the beam degraded to 20.07 MeV when it reached the Pr_6_O_11_ pellet, which was close to the desired 20 MeV beam energy goal. Irradiation at 10 μA for 10 min produced 25.8 ± 3.2 MBq (696.2 ± 88.6 μCi) of ^140^Nd at the end of bombardment (EOB). Increasing the irradiation time and beam current to 4 h and 20 μA, respectively, resulted in a yield of 1417.0 ± 83.4 MBq (38.3 ± 2.2 mCi) at the EOB (Table 1). The γ ray spectrum analysis confirmed the production of ^140^Nd (Figure S2). ^141^Nd and ^139m^Nd decayed to the background by 48 h postirradiation (Figure S2). ^139^Ce was observed during these 4 h-long irradiations at a yield of 4.55 ± 1.13 kBq (0.12 ± 0.03 μCi) at the EOB.

**Table 1:** ^140^Nd Yields at the End of Bombardment for Irradiation of the 100 mg Pr_6_O_11_ Pellet Target at 24 MeV (20.07 MeV at the Pr Surface) at Varying Beam Currents and Irradiation Times

irradiation parameter	experimental activity	theoretical activity	ratio (experimental/theoretical)
10 μA, 10 min	25.8 ± 3.2 MBq (696.2 ± 88.6 μCi)	31.5 MBq (852 μCi)	0.82 ± 0.10
10 μA, 40 min	123.6 MBq (3.3 mCi)	124.8 MBq (3.4 mCi)	0.99
10 μA, 60 min	162.1 MBq (4.4 mCi)	187.2 MBq (5.1 mCi)	0.87
15 μA, 60 min	307.1 MBq (8.3 mCi)	282.7 MBq (7.6 mCi)	1.08
20 μA, 3 h	1036.0 MBq (28.0 mCi)	1121.5 MBq (30.3 mCi)	0.92
20 μA, 4 h	1417.0 ± 83.4 MBq (38.3 ± 2.2 mCi)	1489.2 MBq (40.2 mCi)	0.95 ± 0.06

### ^140^Nd Purification

The optimized method for the purification of ^140^Nd utilized three DGA normal resin columns, containing 4.5 g (column 1), 1.8 g (column 2), and 300 mg (column 3) resin amounts. The irradiated pellet was digested using con. HCl, diluted to 15 mL final volume, and loaded (1-D) into column 1, and flowthrough was collected (1-FT). Column 1 was washed with 2.7 M HCl in four portions (1-W1 (650 mL), 1-W2 (13 mL), 1-W3 (13 mL), 1-W4 (13 mL)) to remove the bulk Pr target material followed by one more wash (1-W5) with 2.2 M HCl (40 mL) to recover the ^140^Nd portion where 88.4 ± 10.9% ^140^Nd was collected. The 1-W1 wash recovered 96.5 ± 1.7% ^141^Pr target material, which was recycled for future use. The ^140^Nd recovered from column 1 in the 1-W5 portion was further purified by loading into column 2 by reformulating the concentration to ∼4 M HCl (50 mL). The loaded ^140^Nd flowthrough from column 2 (2-FT) was collected, and the column was washed with three portions of 3 M HCl as 2-W1 (200 mL), 2-W2 (13 mL), and 2-W3 (13 mL) followed by a ^140^Nd wash (2-W4, 2.2 M HCl, 30 mL), recovering 76.1 ± 4.6% ^140^Nd. This collected ^140^Nd amount was reformulated to >4 M HCl (40 mL) for further purification and to minimize the final ^140^Nd-containing volume using column 3. The flowthrough of loaded ^140^Nd in this column was collected as 3-FT, followed by washing the column with 3 M HCl (40 mL). After washing, the column was eluted in two portions, 3-E1 and 3-E2, each with 400 μL of Milli-Q water. The 3-E2 elution fraction recovered 71.6 ± 6.3% ^140^Nd in a final volume of 400 μL, which was used for downstream studies (Figure 3).

**Figure 3:**
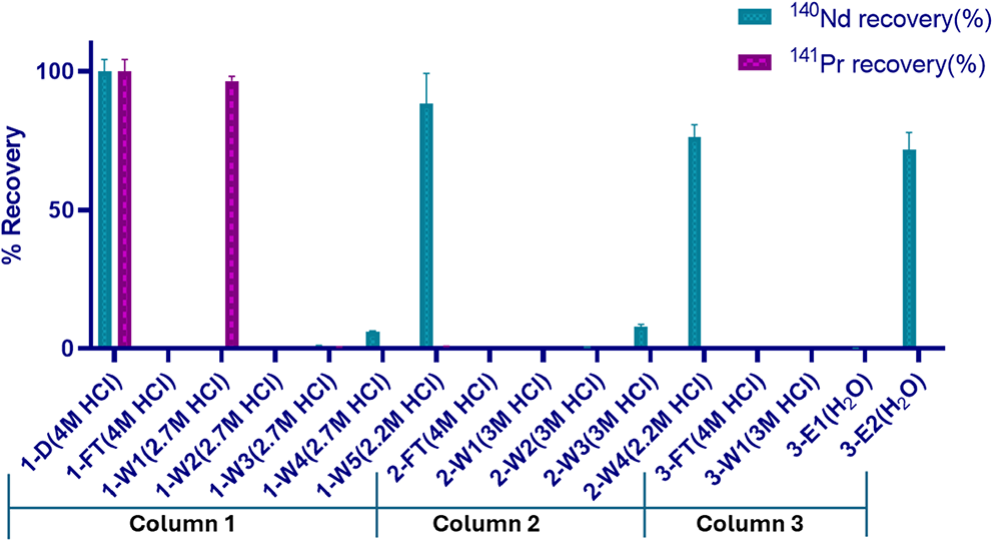
Separation profile of the optimized three-column DGA normal resin method for the purification of ^140^Nd from the ^141^Pr target material.

The concentration of stable Nd in the purified ^140^Nd product decreased progressively with each target material recycling cycle. When the nonrecycled target material was used, the stable Nd concentration was 348,528 ppb. After the third recycling cycle, this value decreased to 226 ppb (Table 2), demonstrating a significant improvement in chemical purity with repeated recycling. Additionally, the concentration of other measured impurities was below 30 ppb in the ^140^Nd sample purified and produced using the third cycle of recycled Pr (Table 2).

**Table 2:** Concentration of Impurities Measured Using ICP-MS in the Purified ^140^Nd Sample Produced Using the Recycled Pr Target Material at Different Stages of Recycling

	impurity concentrations (ppb)			
element	without recycling	Cycle 1	Cycle 2	Cycle 3
Sc	<1	<1	<1	<1
La	<1	<1	<1	<1
Ce	19	1	<1	3
Pr	18	31	<1	4
Nd	348,528	17,584	2085	226
Sm	14	<1	<1	<1
Eu	<1	<1	<1	<1
Gd	<1	<1	<1	<1
Tb	55	5	<1	<1
Dy	281	3	<1	<1
Ho	24	7	<1	<1
Er	<1	<1	<1	<1
Tm	<1	<1	<1	<1
Yb	<1	<1	<1	<1
Lu	<1	<1	<1	<1
Cr	23	2	1	1
Fe	398	43	13	29
Co	<1	<1	<1	<1
Ni	<1	3	<1	3
Cu	51	<1	<1	<1
Pb	42	11	1	1

### Radiolabeling

DOTA and Macropa chelators were radiolabeled with purified ^140^Nd in 1 M NH_4_OAc buffer solution by incubation at 37 °C for 30 min to afford [^140^Nd]­Nd-DOTA and [^140^Nd]­Nd-Macropa and analyzed using radio-TLC. The apparent molar activity (AMA) of both radiolabeled complexes increased substantially with increasing chemical purity of ^140^Nd (Table 3). Using ^140^Nd obtained from the nonrecycled target material, the AMA was 70.3 MBq/μmol (1.9 mCi/μmol) for [^140^Nd]­Nd-DOTA and 74.0 MBq/μmol (2.0 mCi/μmol) for [^140^Nd]­Nd-Macropa. ^140^Nd produced after the third recycling cycle yielded markedly higher AMA values of 8025.3 MBq/μmol (216.9 mCi/μmol) for [^140^Nd]­Nd-DOTA and 8473.0 MBq/μmol (229.0 mCi/μmol) for [^140^Nd]­Nd-Macropa. These results demonstrate that progressive reduction of stable Nd impurities through target recycling significantly enhances achievable molar activity.

**Table 3:** Apparent Molar Activity of [^140^Nd]­Nd-DOTA and [^140^Nd]­Nd-Macropa Labeled with ^140^Nd Produced from Each Cycle of Purified ^141^Pr Target Material

purification cycle	[^140^Nd]Nd-DOTA AMA	[^140^Nd]Nd-Macropa AMA
without recycling	(1.9 mCi/μmol) 70.3 MBq/μmol	(2.0 mCi/μmol) 74 MBq/μmol
Cycle 1	(9.5 mCi/μmol) 351.5 MBq/μmol	(7.6 mCi/μmol) 281.2 MBq/μmol
Cycle 2	(30.3 mCi/μmol) 1121.1 MBq/μmol	(49.5 mCi/μmol) 1831.5 MBq/μmol
Cycle 3	(216.9 mCi/μmol) 8025.3 MBq/μmol	(229 mCi/μmol) 8473 MBq/μmol

### In Vitro Stability Studies

The stability of [^140^Nd]­Nd-DOTA and [^140^Nd]­Nd-Macropa was evaluated in saline (0.9% w/v), phosphate-buffered saline (PBS), mouse serum, and human serum at a 10-fold dilution, with incubation at 37 °C for up to 5 days (Figure 4). [^140^Nd]­Nd-DOTA remained intact throughout the 5-day study period, maintaining >95% intact complex in all tested media (Figure 4a). [^140^Nd]­Nd-Macropa also demonstrated >95% stability in saline, PBS, and mouse serum over 5 days. However, in human serum, the intact complex decreased over time, reaching 80.3 ± 0.9% intact complex on day 5 (Figure 4b).

**Figure 4:**
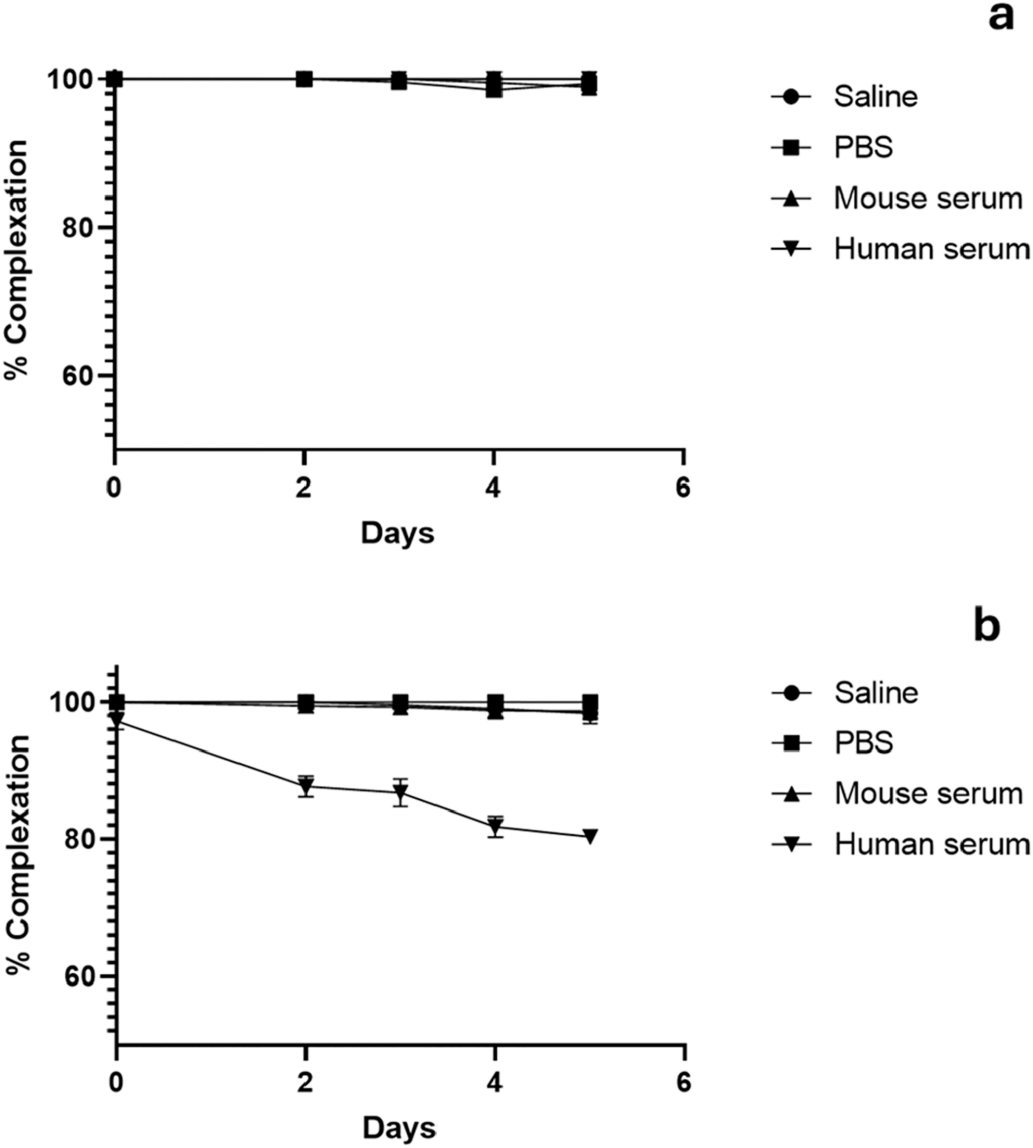
Stability of ^140^Nd-radiolabeled complexes in saline (0.9% w/v), PBS, mouse serum, and human serum tested in 10 times the volume of the reaction mixture at 37 °C. (a) Stability of [^140^Nd]­Nd-DOTA. (b) Stability of [^140^Nd]­Nd-Macropa.

### Free ^140^Nd Protein Binding in Blood

Free ^140^Nd (0.93 MBq; 25 μCi) was incubated with freshly collected mouse blood (1 mL) at 37 °C, and its distribution among plasma protein, free plasma, and red blood cell (RBC) fractions was analyzed at 30 min, 1, 2, and 5 h (Figure 5). At 30 min, 72.7% of the total ^140^Nd activity was associated with the plasma protein fraction. This value increased slightly to 76.2% after 5 h of incubation at 37 °C. In contrast, ^140^Nd associated with the RBC fraction decreased modestly from 27.3% at 30 min to 23.8% at 5 h. No measurable activity was detected in the free plasma fraction at any time point during the 5 h incubation period.

**Figure 5:**
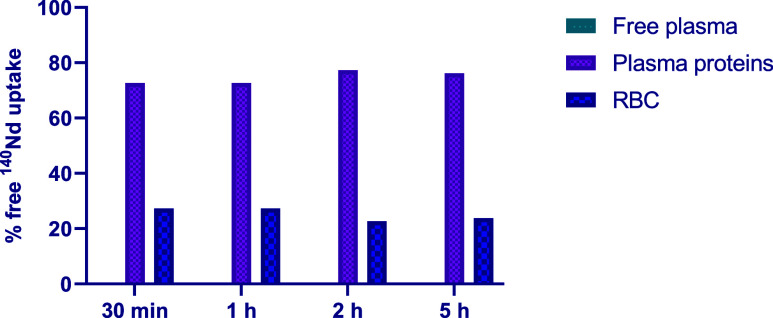
Free ^140^Nd uptake in mouse blood fractions in freshly pooled blood and incubated at 37 °C.

## Discussion

The Pr target material was recovered as praseodymium oxide from the recycling process. TGA analysis reveals three steps of weight reductions upon heating up to 800 °C, turning Pr­(OH)_3_ to praseodymium oxides.^24^ The Pr and O atomic % in SEM-EDX elemental analysis of the praseodymium oxide pellet surface reveals the existence of multiple oxide forms of Pr. The Cl content can be attributed to residual chlorides such as PrCl_3_ that may have remained from the neutralization process. The thermal treatment was intended to obtain the recovered Pr in an oxide form suitable for pellet fabrication and irradiation. For this work, we referred to all the oxide forms as Pr_6_O_11_ as it is the likely species based on the literature precedent for thermal conversion of the praseodymium hydroxide system under applied heating conditions and the appearance of the final oxide form in dark brown color.^24^ The pellet synthesized from Pr_6_O_11_ remains intact during the tested irradiation time windows up to 4 h, which makes it a suitable material to achieve the ^140^Nd yields required for preclinical applications and potential future clinical applications, as well as for distribution.

The beam interaction at the surface of the Pr_6_O_11_ pellet was degraded to 20.07 MeV using an Al degrader. Crossing through the pellet, the beam energy from 20.07 to 18.18 MeV captures the peak (18−24 MeV) of the reaction cross section of ^141^Pr­(p,2n)^140^Nd, while producing a minimal amount of side products. At 4 h irradiation using 20 μA beam current, the produced ^140^Nd was 17.71 ± 1.04 MBq/μAh (0.479 ± 0.027 mCi/μAh) at the EOB. In comparison, Zhernosekov et al. reported 15.5 MBq/μAh (0.419 mCi/μAh) by irradiating a 200 mg praseodymium oxide pellet, Aboudzadeh et al. reported 240.5 MBq/μAh (6.5 mCi/μAh) using a 1.1 mm-thick praseodymium oxide pellet, and our previous work reported 13.67 MBq/μAh (0.369 mCi/μAh) by irradiating a 0.1 mm-thick Pr foil (∼50 mg).^10,23,25^ At the irradiated proton energy, side reactions, including ^141^Pr­(p,n)^141^Nd (*t*_1/2_: 2.5 h), ^141^Pr­(p,3n)^139m^Nd (*t*_1/2_: 5.5 h), and ^141^Pr­(p,x)^139^Ce (*t*_1/2_: 137.6 d), may occur. The γ-ray spectrum measured 48 h after the EOB reveals that these impurities are minimal (Figure S2) with the exception of long-lived ^139^Ce (*t*_1/2_: 137.6 d, 4.55 ± 1.13 kBq (0.12 ± 0.03 μCi) upon 4 h, 20 μA irradiation). The commercially sourced ^141^Pr target contained around 1% Nd and Gd each among other impurities (Table S1). There are certain possible reactions at the proton energy of 20 MeV, including ^Nat^Nd­(p,x)^143,144,146,148,48m,149,150^Pm, ^Nat^Nd­(p,x)^142^Pr, ^Nat^Nd­(p,x)^141,147,149^Nd, and ^Nat^Gd­(p,x)^151,152,153,154m,155,156,160^Tb, when protons interact with these impurities.^26,27^ However, products of these reactions were minimal and not observed with the purified material.

Because praseodymium (Pr) and neodymium (Nd) are adjacent lanthanides, their chemical properties are highly similar, making their separation particularly challenging. To address this, a solid-phase extraction method using DGA normal resin was developed to effectively separate ^140^Nd from ^141^Pr. This resin contains embedded *N*,*N*,*N*′,*N*′-tetraoctyldiglycolamide groups, which interact with the lanthanide to form complexes of M­(DGA)_3_Cl_3_ through equilibrium M^3+^ + 3DGA + 3Cl^−^. At high concentrations of the HCl mobile phase, the lanthanide ions are retained on the resin, whereas at lower concentrations, they are more readily eluted, thus creating a concentration-based resin affinity reflected as a weight distribution constant (*D*_w_). The normal DGA resin has a Nd/Pr separation factor of 2.5, the highest among LN, LN2, LN3, and branched DGA resins, and weight distribution constants (*D*_w_) of ∼12 for Pr and ∼30 for Nd in 3 M HCl as reported by the manufacturer. Compared with our previous report, which used 2.5 M HCl, the use of 2.7 M HCl in column 1 and 3 M HCl in column 2 provided improved separation of the Nd and Pr elution bands. This resulted in more effective removal of bulk Pr while retaining and subsequently eluting ^140^Nd with improved purity and higher recovery of 71.6 ± 6.3% after a three-column separation. This increase in ^140^Nd recovery from ∼27% in our previous work to ∼71% in the present study is attributable to optimization of the purification process, including implementation of the three-column DGA workflow and improved control of acid concentration, elution volumes, and fractionation.^10^

Our previous report on ^140^Nd production via the foil method and the development of a separation method established a robust route for acquiring ^140^Nd.^10^ However, the presence of stable Nd in the target material compromised the radiochemical purity of purified ^140^Nd, which is key for applications in PET imaging. Additionally, the postpurification recovery of ^140^Nd was below 30%. The adjacent lanthanides, which have similar chemical properties, pose a significant challenge in separating the Nd from the Pr target material. Additionally, unlike Ce/Nd, where Ce can achieve a +4 oxidation state, thus creating a chemical difference with Nd^3+^, as was utilized by Rosch et al., in the case of Pr/Nd, an alternate oxidation state for either is unstable for utilization during the process.^11^ In this work, the development of target recycling purified the target material, and optimization of the separation method increased the recovery yield of ^140^Nd. The cumulative results of both processes increased the radiochemical purity of ^140^Nd. The stable Nd content in purified ^140^Nd decreased by more than 1500 times, with other measured stable metal impurities decreased below 30 ppb. In the final purified ^140^Nd, the presence of the target material Pr itself remains below 35 and 5 ppb when using the third cycle of the recycled target material as a result of the optimized separation process, having minimal impact in downstream studies pertaining to the purity of ^140^Nd. Overall, the reduction in these impurities was reflected in the increase >100 times in apparent molar activity of both [^140^Nd]­Nd-DOTA and [^140^Nd]­Nd-Macropa. To the best of our knowledge, this work is the only report for ^140^Nd production via a medium-energy cyclotron route for pure ^140^Nd for use in imaging applications.

As Nd precipitates above pH 3 in solutions such as saline and water, to avoid undesirable results, ^140^Nd must be used below pH 3 or stabilized above pH 3 by using coordinating buffers such as ammonium acetate or citrate.

One of the challenges of an in vivo PET generator is the impact of dechelation of the daughter. For ^140^Nd-labeled radiotracers, it is the daughter radionuclide, ^140^Pr, that emits the positron for PET imaging. However, decay recoil and/or electron ionization may cause dissociation of the daughter from the parent chelate, leaving a free ^140^Pr daughter in the surrounding. Bauer et al., Severin et al., Bobba et al., and Bailey et al. have highlighted the impact of daughter radionuclide redistribution on the accuracy of PET imaging with in vivo PET generators.^28–31^ This dechelation effect also poses a challenge during radiochemical analysis when radio-TLC and radio-HPLC are used. In radio-TLC, the dechelated free ^140^Pr migrates at a different rate than the ^140^Nd-labeled compound during the plate development, thereby disrupting the ^140^Nd–^140^Pr secular equilibrium and potentially giving misleading results. Hence, all of the radio-TLC plates were analyzed after a minimum of 45 min following the TLC development, allowing sufficient time for equilibrium to be re-established. Similarly, in radio-HPLC, the continuous flow of the mobile phase disrupts the ^140^Nd–^140^Pr secular equilibrium. Because radio-HPLC measures radioactivity in real time, the resulting chromatogram reflects this disrupted equilibrium and therefore does not provide an accurate assessment of the radiochemical yield. Hence, radio-TLC was used for the radiochemical analysis of the ^140^Nd-labeled compounds.

While [^140^Nd]­Nd-DOTA was stable in saline, PBS, mouse serum, and human serum, [^140^Nd]­Nd-Macropa illustrated instability in human serum. This reduced stability of [^140^Nd]­Nd-Macropa in human serum is likely due to differences in the composition including serum proteins and the metal-binding components of human serum relative to mouse serum. These species are absent in PBS and saline and may differ sufficiently between human and mouse sera to influence complex stability. Similarly, the difference in the stability of [^140^Nd]­Nd-DOTA compared to [^140^Nd]­Nd-Macropa reveals the role of the chelator structure. DOTA has four pedantic arms that can stabilize the metal ion in the vicinity of the chelator; similarly, the core is smaller compared to Macropa. Nd-DOTA adopts a square antiprismatic structure with all the pedantic arms on one side of the core, stabilizing the Nd in the vicinity.^32^ In comparison, Macropa has only two pedantic arms and a bigger core structure compared to DOTA. Although the crystal structure of Nd-Macropa is not yet established, Roca-Sabio et al., Thiele et al., and Hu et al. have highlighted the decreasing thermodynamic stability of lanthanide-Macropa complexes from La^3+^ to Lu^3+^.^33–35^ Several in vivo investigations employing Macropa-conjugated tracers labeled with the early lanthanides ^132/133^La and ^134^Ce have demonstrated favorable serum and in vivo stability, suggesting the role of ionic size in the formation of stable Macropa complexes.^30,31,36,37^ Overall, early lanthanides make stable Macropa complexes compared to later lanthanides, and the reduced [^140^Nd]­Nd-Macropa stability suggests that Nd might be an inflection point between stable and unstable lanthanide-Macropa complexes for the lanthanides.

The in vivo PET generator neodymium-140 (^140^Nd (*t*_1/2_: 3.4 d, EC))/praseodymium-140 (^140^Pr (*t*_1/2_: 3.4 min, 51.0% β^+^)) holds the potential to be used as an imaging surrogate for f-block therapeutic nuclides and potentially exhibit close pharmacokinetics due to similar chemical properties.^28,29^ In the f-block series, ^132^La (*t*_1/2_: 4.8 h, 42.1% β^+^) and ^133^La (*t*_1/2_: 3.9 h, 7.1% β^+^) have been studied as PET imaging theranostic counterparts to the α-emitter ^225^Ac (*t*_1/2_: 9.9 d) and Auger electron emitter ^135^La (*t*_1/2_: 19.5 h) from production to preclinical studies by several groups.^37–43^ A recent study carried out by Aluicio-Sarduy et al. used ^132^La and ^225^Ac to radiolabel an alkylphosphocholine analogue (NM600), and this theranostic pair revealed comparative biodistribution behavior.^38^ Similarly, preclinical evaluation of [^132/135^La]­La-FAPI-2286 by Shirpour et al. and [^133^La]­La-PSMA-I&T by Nelson et al. revealed high cellular and tumor uptake.^44,45^ Recently, the ^134^Ce/^134^La in vivo PET generator has been explored by a few groups via ^134^Ce-labeled targeting vectors. For example, [^134^Ce]­Ce-Macropa-PEG4-YS5, [^134^Ce]­Ce-DOTA-trastuzumab, and [^134^Ce]­Ce-PSMA-617 were studied in combination with ^225^Ac as theranostic matches for targeted α-therapy studies and [^134^Ce]­Ce-cDTPA-ATN-291 for exploratory PET imaging studies.^28–31,46^ Although much further studies are required, these studies suggest that ^134^Ce/^134^La illustrates similar results to those with ^225^Ac. Clinical studies using [^152^Tb]­Tb-DOTANOC and [^152^Tb]­Tb-PSMA-617 were performed using ^152^Tb produced from ISOLDE-CERN via spallation processes.^47^ Although PET imaging demonstrated increased noise due to higher positron energy and photon coemissions, the relatively long half-life enabled imaging at extended time points (up to 24−25 h postinjection), allowing detection of small metastases. Despite these advantages, production of these radionuclides is a major challenge due to either the requirement of an expensive enriched material and high-energy proton beam energy or both.^48,47^ Other challenges including coemission of γ radiation, short half-life, and moderate positron branching ratio limit broader adoption of these radionuclides. Alternatively, ^140^Nd production can be accessed at lower proton energies that are available with medium-energy cyclotrons and requires a monoisotopic ^141^Pr target material.^10^

The ^140^Nd/^140^Pr in vivo PET generator is a highly underexplored system. The studies carried out in this manuscript are part of a pipeline that addresses several key production and purification barriers, hence serving to support a broader goal of establishing the ^140^Nd/^140^Pr in vivo generator system as a robust PET analogue. The in vitro studies including ^140^Nd-chelator stability and free ^140^Nd blood uptake provide important reference points to this goal. Future studies will explore the ^140^Nd/^140^Pr in vivo generator with a variety of chelator systems and preclinical imaging studies using ^140^Nd-labeled targeting molecules including antibodies and peptides on xenograft mouse models.

## Conclusions

We report a robust and scalable approach for producing high-purity ^140^Nd via the ^141^Pr­(p,2n)^140^Nd reaction by integrating oxide targetry, target recycling, and an optimized multicolumn DGA purification strategy. Progressive reduction of stable Nd impurities through target recycling enabled a dramatic increase in apparent molar activity (>8 GBq/μmol (>200 mCi/μmol)), overcoming the carrier-added limitations in previous reports. Radiolabeling of DOTA and Macropa confirmed efficient complexation of purified ^140^Nd. Together, these advances establish a practical route to high-molar activity ^140^Nd and strengthen the foundation for further development of the ^140^Nd/^140^Pr in vivo generator system, supporting the overall goal of development as a robust PET imaging surrogate suitable for translational molecular imaging applications specifically for f-block therapeutics.

## Methods

### Materials and Instrumentation

Praseodymium foil (99.8% purity), tantalum sheets (3N8 purity), and aluminum sheets (3N purity) were purchased from ESPI Metals (Ashland, OR, USA). Normal DGA (*N*,*N*,*N*,,*N*′-tetra-*n*-octyldiglycolamide) resin (50−100 μm particle size) was obtained from Eichrom Technologies (Lisle, IL, USA). Polypropylene solid-phase extraction (SPE) columns (1 mL) were purchased from MilliporeSigma (Supelco, Sigma-Aldrich, St. Louis, MO, USA). PEEK empty HPLC columns (7.4 mm × 300 mm and 4.6 mm × 300 mm) were obtained from Analytics-Shop USA LP (Stockbridge, GA, USA). The Macropa chelator (2-pyridinecarboxylic acid, 6,6′-[1,4,10,13-tetraoxa-7,16-diazacyclooctadecane-7,16-diylbis­(methylene)]­bis-) was purchased from MedChemExpress (Monmouth Junction, NJ, USA), and DOTA (1,4,7,10-tetraazacyclododecane-1,4,7,10-tetraacetic acid) was obtained from Macrocyclics (Plano, TX, USA). Paper-backed silica gel iTLC-SG plates were purchased from Agilent Technologies (Santa Clara, CA, USA). All other chemicals were purchased from Sigma-Aldrich or Fisher Scientific and were trace metal grade unless otherwise stated. Praseodymium oxide pellets were fabricated using a hydraulic press (Carver Inc., Wabash, IN, USA) and a 10 mm-inner diameter dry pellet pressing die set. Stable elemental analysis and quantification were performed by inductively coupled plasma mass spectrometry (ICP-MS; Agilent 7900, Agilent Technologies, Santa Clara, CA, USA) using multielement calibration standards obtained from Agilent Technologies. γ-ray spectroscopy for radionuclide identification, yield determination, and radionuclidic purity assessment was conducted using a calibrated high-purity germanium detector (HPGe; Canberra GC2018) interfaced with a DSA-100 multichannel analyzer (Mirion Technologies, Meriden, CT, USA), with spectra analyzed using Genie 2000 software (Mirion Technologies, Meriden, CT, USA). A dose calibrator (CRC-25R, Mirion Technologies, Florham Park, NJ, USA), cross-calibrated against the HPGe system, was used for radioactivity measurements throughout separation, radiolabeling, and imaging studies. Radiochemical yields of ^140^Nd-labeled compounds were determined using a radio-TLC scanner (AR-2000, Eckert & Ziegler, Berlin, Germany).

### Target Purification and Recycling

A target recycling method was developed to purify the ^141^Pr target material. Praseodymium foils (thickness 0.1 mm, diameter 10 mm) were digested in 4 M HCl and allowed to cool to room temperature. For the removal of stable neodymium impurities, a reusable column (PEEK, 7.4 mm × 300 mm) packed with 4.5 g of DGA normal resin was employed. The digested ^141^Pr solution (15 mL, containing ∼100 mg of Pr) was loaded onto the column at a flow rate of 1 mL/min. Separation of Pr and Nd was achieved by washing the column with 450 mL of 2.7 M HCl at 1 mL/min to elute the Pr fraction. Subsequently, the column was rinsed with 20 mL of 0.1 M HCl to remove the remaining retained species, including stable Nd. Sodium hydroxide pellets were added to the collected Pr fraction in an ice bath until the pH reached ≥8, resulting in the precipitation of Pr as Pr­(OH)_3_. The suspension was vacuum-filtered through a 0.22 μm nitrocellulose membrane, washed with Milli-Q water (18.2 MΩ-cm), and dried overnight. Thermogravimetric analysis (TGA) of the dried Pr­(OH)_3_ was performed by heating a small amount (∼4 mg) from 30 to 800 °C at a rate of 5 °C/min. Bulk Pr­(OH)_3_ was heated at 800 °C for 24 h to obtain Pr_6_O_11_. The resulting oxide was ground to a fine powder using a mortar and pestle and heated at 200 °C for 30 min to remove residual moisture prior to making a pellet. Approximately 100 mg of Pr_6_O_11_ was pressed into a pellet using a 10 mm-inner diameter dry die press under stepwise increasing pressure. The sample was first pressed at 2 t for 15 min, followed by 4 t for an additional 15 min, and finally at 6 t for 10 min. The pellet was dissolved in concentrated HCl at 180 °C for 30 min, completing one target purification cycle. Stable elemental concentrations were quantified by ICP-MS. For surface morphology and elemental composition analysis of the Pr_6_O_11_ pellet, a thin layer of gold was sputter-coated onto the pellet surface and analyzed using scanning electron microscopy (SEM) with energy dispersive X-ray spectroscopy (EDS).

### Target Irradiation

The target assembly for ^140^Nd production consisted of a 0.75 mm-thick (Al) degrader (on the incident beam side), followed by a 0.34 mm-thick and 10.21 mm-diameter Pr_6_O_11_ pellet, which was inserted into a circular tantalum (Ta) backing with a divot (10.22 mm diameter, 0.80 mm depth) in the middle and 23.70 mm overall diameter and 1.44 mm overall thickness (Figure 6). The target was irradiated with a 24 MeV proton beam at UAB’s TR24 cyclotron. Proton energy degradation and the beam interaction with the target were modeled using SRIM-2013. Beam currents and irradiation times from 10 μA and 10 min to 20 μA and 4 h, respectively, were tested in increasing order to achieve preclinically relevant yield while maintaining pellet integrity (Table 1). The irradiated pellet was digested in conc. HCl with heating at 180 °C until evaporation. The residue was reconstituted in 15 mL of 4 M HCl, and aliquots were collected for radionuclidic analysis and radioactivity quantification using γ-ray spectroscopy.

**Figure 6:**
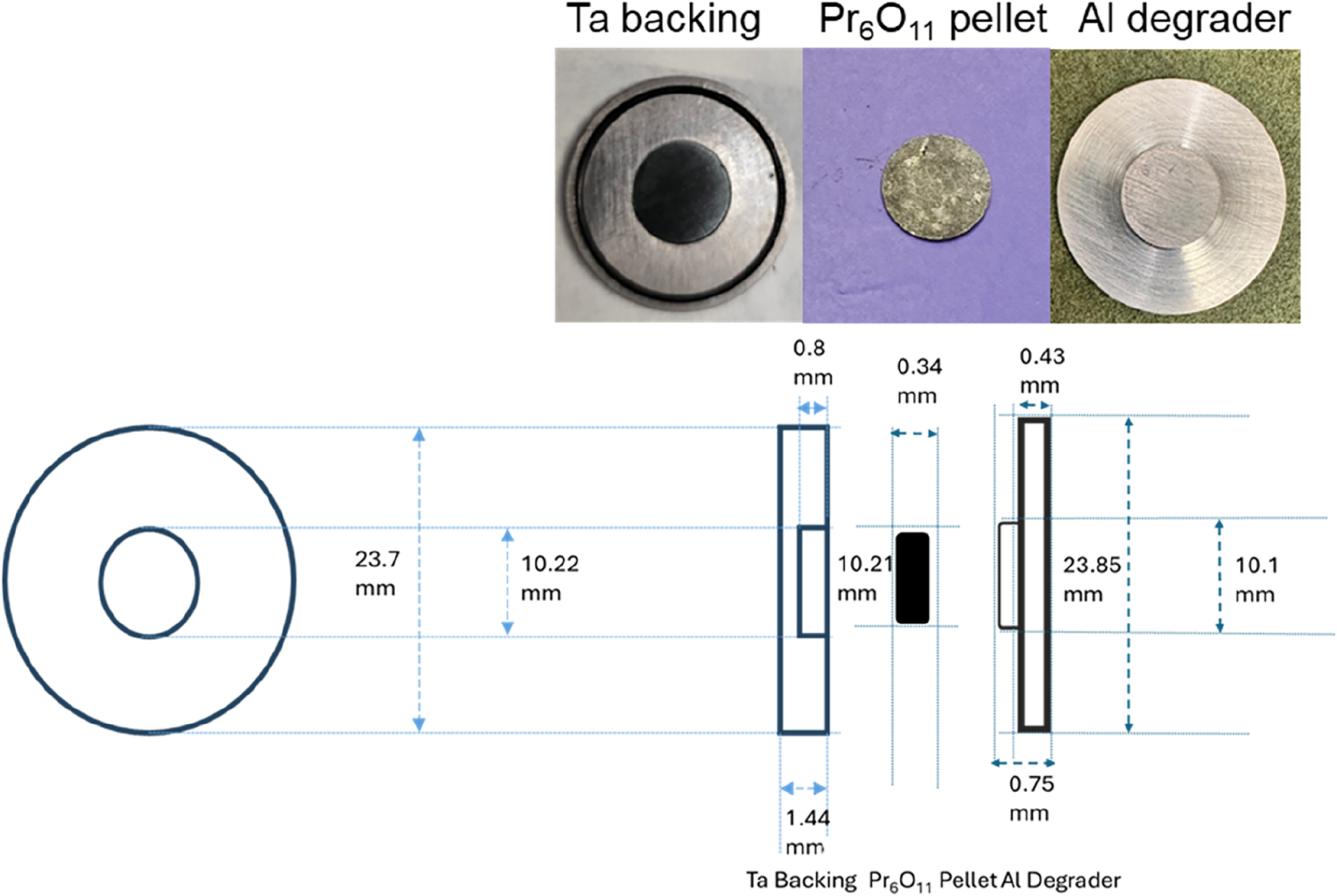
Target assembly of the Pr_6_O_11_ pellet for irradiation at the cyclotron.

### ^140^Nd Purification

Neodymium−praseodymium lanthanide separation remains challenging and was previously reported by our group with an overall ^140^Nd recovery of approximately 27%.^10^ The present work aimed to improve both the recovery yield and chemical purity of ^140^Nd through optimization of a three-column separation strategy using DGA normal resin.

Column 1: A PEEK column (7.4 mm × 300 mm) was packed with 4.5 g of DGA normal resin and preconditioned with 15 mL of 0.1 M HCl, followed by 15 mL of 4 M HCl. The irradiated and digested pellet solution (1-D; 15 mL, 4 M HCl) was loaded at 1 mL/min, and the flowthrough (1-FT; 15 mL, 4 M HCl) was collected. The column was then washed with 2.7 M HCl in four fractions of 650 mL (1-W1), 13 mL (1-W2), 13 mL (1-W3), and 13 mL (1-W4) to remove the bulk Pr target material. Subsequently, ^140^Nd was eluted by using 2.2 M HCl (40 mL, 1-W5). The column was rinsed with 0.1 M HCl (20 mL, 1-W6) and stored for reuse.

Column 2: A second PEEK column (4.6 mm × 300 mm) containing 1.8 g of DGA normal resin was preconditioned in the same conditions as column 1. The ^140^Nd-containing fraction from column 1 (1-W5) was loaded onto column 2 after 10 mL of con. HCl, with the flowthrough collected (2-FT; 50 mL). The column was washed with 3 M HCl in three fractions of 200 mL (2-W1), 13 mL (2-W2), and 13 mL (2-W3). ^140^Nd was then eluted with 2.2 M HCl (30 mL, 2-W4). The column was rinsed with 0.1 M HCl (20 mL) and stored for reuse.

Column 3: The ^140^Nd fraction from column 2 (2-W4) was reformulated to >4 M HCl by adding con. HCl and further purified using a third column consisting of 300 mg of DGA normal resin packed in a 1 mL SPE cartridge. The loaded solution was collected as flowthrough (3-FT; 40 mL). The column was washed with 40 mL of 3 M HCl (3-W1) and eluted in two fractions with Milli-Q water (18.2 MΩ-cm) (400 μL, 3-E1 and 3-E2, 0.4 mL/min). The final purified ^140^Nd was collected in fraction 3-E2 and used for subsequent studies.

Aliquots from all collected fractions were analyzed for radionuclidic composition by HPGe γ-ray spectroscopy and for stable elemental content by ICP-MS.

### Radiolabeling and Stability Studies

DOTA and Macropa chelators were radiolabeled with ^140^Nd following a previously reported method (Godara et al.).^10^ Briefly, serial dilutions of Macropa and DOTA were prepared in 1 M ammonium acetate buffer (NH_4_OAc, pH 6.5) in microcentrifuge tubes. Approximately 1.85 MBq (50 μCi) of ^140^Nd was added to each tube, and the reactions were incubated at 37 °C for 30 min. Radiochemical yields were determined by radio-TLC. Aliquots of the reaction mixtures were spotted onto silica gel-impregnated paper plates and developed using 0.1 M citric acid as the mobile phase. The developed plates were analyzed using a radio-TLC scanner, and yields were quantified using WinScan software (Eckert & Ziegler, Berlin, Germany). Apparent molar activity was calculated by dividing the added ^140^Nd activity by twice the molar amount of chelator corresponding to 50% radiochemical yield. The labeled complexes, [^140^Nd]­Nd-DOTA and [^140^Nd]­Nd-Macropa, were evaluated for in vitro stability in phosphate-buffered saline (PBS), saline (0.9% w/v), mouse serum, and human serum. For each condition, 30 μL of the radiolabeled reaction mixture was added to 300 μL of the respective medium and incubated at 37 °C for up to 5 days.

### Free ^140^Nd Protein Binding in Blood

Whole blood (1 mL) was collected from a mouse under 2.5% inhaled isoflurane anesthesia in oxygen at 2 L/min using a heparin-rinsed syringe and transferred to a microcentrifuge tube. Free ^140^Nd (0.93 MBq; 25 μCi) was added to the blood sample and incubated at 37 °C and 600 rpm on a thermomixture. At predetermined time points (30 min, 1, 2, and 5 h), 200 μL aliquots of the blood mixture were collected. Plasma proteins, free plasma, and red blood cell (RBC) fractions were separated using a standard blood fractionation protocol. Briefly, the blood sample was centrifuged at 2000*g* for 10 min to separate plasma and RBCs. The plasma (upper layer) was transferred to a new tube. 2-fold volumes of cold methanol were added to the plasma, followed by vortex and centrifugation at 14,000*g* for 2 min. The resulting supernatant (free plasma) fraction was collected in a separate tube, while the remaining pellet contained plasma proteins. The radioactivity associated with each fraction was measured using a Hidex automatic γ counter.

## Supplementary Material



## Notes

Stable impurities present in the commercially sourced ^141^Pr metal foil, γ-ray spectrum of the irradiated target, and additional experimental details on the weight distribution coefficient (*D*_w_): methods and results (PDF)

## References

[1] Lapi S. E., Scott P. J. H. (2024). The future of
the radiopharmaceutical sciences. Nucl. Med.
Biol..

[2] Herrmann K., Schwaiger M., Lewis J. S., Solomon S. B., McNeil B. J., Baumann M., Gambhir S. S., Hricak H., Weissleder R. (2020). Radiotheranostics:
a roadmap for future development. Lancet Oncol..

[3] Lapi S. E., Scott P. J. H., Scott A. M., Windhorst A. D., Zeglis B. M., Abdel-Wahab M., Baum R. P., Buatti J. M., Giammarile F., Kiess A. P. (2024). Recent advances and
impending challenges for the radiopharmaceutical sciences in oncology. Lancet Oncol..

[4] Rosar F., Burgard C., Petrescu C., Blickle A., Bartholomä M., Maus S., Bastian M. B., Speicher T., Schaefer-Schuler A., Ezziddin S. (2025). Pilot experience of
[^161^Tb]­Tb-PSMA-617 RLT
in mCRPC patients after conventional PSMA RLT within a prospective
registry. Theranostics.

[5] Strosberg J., El-Haddad G., Wolin E., Hendifar A., Yao J., Chasen B., Mittra E., Kunz P. L., Kulke M. H., Jacene H. (2017). Phase 3 Trial of 177Lu-Dotatate for Midgut Neuroendocrine
Tumors. N. Engl. J. Med..

[6] Nelson B. J. B., Andersson J. D., Wuest F. (2021). Targeted Alpha Therapy: Progress
in Radionuclide Production, Radiochemistry, and Applications. Pharmaceutics.

[7] Sartor O., Bono J. D., Chi K. N., Fizazi K., Herrmann K., Rahbar K., Tagawa S. T., Nordquist L. T., Vaishampayan N., El-Haddad G. (2021). Lutetium-177−PSMA-617
for Metastatic Castration-Resistant Prostate Cancer. N. Engl. J. Med..

[8] Weineisen M., Schottelius M., Simecek J., Baum R. P., Yildiz A., Beykan S., Kulkarni H. R., Lassmann M., Klette I., Eiber M. (2015). 68Ga- and 177Lu-Labeled PSMA I&T: Optimization
of a PSMA-Targeted Theranostic Concept and First Proof-of-Concept
Human Studies. J. Nucl. Med..

[9] Kurz S. C., Zan E., Cordova C., Troxel A. B., Barbaro M., Silverman J. S., Snuderl M., Zagzag D., Kondziolka D., Golfinos J. G. (2024). Evaluation of the SSTR2-targeted Radiopharmaceutical
177Lu-DOTATATE and SSTR2-specific 68Ga-DOTATATE PET as Imaging Biomarker
in Patients with Intracranial Meningioma. Clin.
Cancer Res..

[10] Godara M. L., Cingoranelli S. J., Tekin V., Saini S., Samuel S. L., Houson H. A., Lapi S. E. (2025). Production and radiochemistry
of
the in vivo PET generator 140Nd/140Pr as an imaging surrogate for
F-block therapeutic radionuclides. Sci. Rep..

[11] Rösch F., Brockmann J., Lebedev N. A., Qaim S. M. (2000). Production and Radiochemical
Separation of the Auger Electron Emitter 140Nd. Acta Oncol..

[12] Severin G. W., Kristensen L. K., Nielsen C. H., Fonslet J., Jensen A. I., Frellsen A. F., Jensen K. M., Elema D. R., Maecke H., Kjær A. (2017). Neodymium-140 DOTA-LM3: Evaluation of an In
Vivo Generator for PET with a Non-Internalizing Vector. Front. Med..

[13] Aikawa M., Hanada Y., Huang H., Haba H. (2021). Activation cross sections
of proton-induced reactions on praseodymium up to 30 MeV. Nucl. Instrum. Methods Phys. Res., Sect. B.

[14] Hilgers K., Shubin Y. N., Coenen H. H., Qaim S. M. (2005). Experimental measurements
and nuclear model calculations on the excitation functions of natCe­(3He,
xn) and 141Pr­(p, xn) reactions with special reference to production
of the therapeutic radionuclide 140Nd. Radiochim.
Acta.

[15] Steyn G. F., Vermeulen C., Nortier F. M., Szelecsényi F., Kovács Z., Qaim S. M. (2006). Production of no-carrier-added 139Pr
via precursor decay in the proton bombardment of natPr. Nucl. Instrum. Methods Phys. Res., Sect. B.

[16] Mukhammedov S., Vasidov A., Pardaev É. (1984). Use
of proton and deuteron activation
methods of analysis in the determination of elements with Z > 42. Sov. At. Energy.

[17] Olkowsky J., Et M. L. P., Gratot I. (1961). Fonction d’excitation
de 141Pr
(p, n) 141Nd Jusqu’à 11.1 MeV. Nucl. Phys..

[18] Becker, D. W. ; Zeisler, S. K. In Measurement of Excitation Functions Using a Pellet Method: Cross-Sections for 140Ce(p,xn), Proc. 8th Int. Workshop on Targetry and Target Chemistry; St. Louis, MO, USA, 1999.

[19] Zeisler S. K., Becker D. W. (1999). Production of the 140Nd/140Pr Radionuclide Generator
for Biomedical Studies. J. Labelled Comp. Radiopharm..

[20] Zeisler, S. K. ; Becker, D. W. In Tumor Imaging with PET Using the Short-Lived Praseodymium-140 from the 140Nd/140Pr In Vivo Generator, 3rd International Conference on Isotopes (3ICI); Vancouver, BC, Canada, 1999.

[21] Zeisler, S. K. ; Becker, D. W. ; Gerlach, L. ; Sinn, H.-J. In Der 140Nd/140Pr In Vivo Radionuklid Generator: Ein Neues Langlebiges Nuklidsystem für PET, Deutsche Gesellschaft für Nuklearmedizin; Munich, Germany, 2000.

[22] Hogan J. J. (1971). Study of
the 141Pr­(p,xn) reaction from 10−85 MeV. J. Inorg. Nucl. Chem..

[23] Zhernosekov K. P., Filosofov D. V., Qaim S. M., Rösch F. (2007). A140Nd/140Pr
radionuclide generator based on physico-chemical transitions in 140Pr
complexes after electron capture decay of 140Nd-DOTA. Radiochim. Acta.

[24] Yan L., Yu R., Liu G., Xing X. (2008). A facile template-free
synthesis
of large-scale single crystalline Pr­(OH)­3 and Pr6O11 nanorods. Scr. Mater..

[25] Aboudzadeh M., Fazaeli Y., Khodaverdi H., Afarideh H. (2012). Production, nano-purification,
radiolabeling and biodistribution study of [140Nd] 5,10,15,20-tetraphenylporphyrin
complex as a possible imaging agent. J. Radioanal.
Nucl. Chem..

[26] Tárkányi F., Hermanne A., Ditrói F., Takács S. (2017). Activation
cross sections of proton induced nuclear reactions on neodymium up
to 65 MeV. J. Radioanal. Nucl. Chem..

[27] Pyles J. M., Irvin T. E., Renne A., Happel S., Nolen J., Lapi S. E. (2025). Production and Purification of Terbium-155 Using Natural
Gadolinium Targets. ACS Omega.

[28] Severin G. W., Fonslet J., Kristensen L. K., Nielsen C. H., Jensen A. I., Kjær A., Mazar A. P., Johnston K., Köster U. (2022). PET in vivo
generators 134Ce and 140Nd on an internalizing monoclonal antibody
probe. Sci. Rep..

[29] Bailey T. A., Wacker J. N., An D. D., Carter K. P., Davis R. C., Mocko V., Larrabee J., Shield K. M., Lam M. N., Booth C. H., Abergel R. J. (2022). Evaluation
of 134Ce as a PET imaging
surrogate for antibody drug conjugates incorporating 225Ac. Nucl. Med. Biol..

[30] Bauer D., De Gregorio R., Pratt E. C., Bell A., Michel A., Lewis J. S. (2024). Examination
of the PET in vivo generator 134Ce as a
theranostic match for 225Ac. Eur. J. Nucl. Med.
Mol. Imaging.

[31] Bobba K. N., Bidkar A. P., Meher N., Fong C., Wadhwa A., Dhrona S., Sorlin A., Bidlingmaier S., Shuere B., He J. (2023). Evaluation of^134^Ce/^134^La as a PET Imaging Theranostic Pair for^225^Ac α-Radiotherapeutics. J. Nucl. Med..

[32] Benetollo F., Bombieri G., Calabi L., Aime S., Botta M. (2003). Structural
Variations Across the Lanthanide Series of Macrocyclic DOTA Complexes:
Insights into the Design of Contrast Agents for Magnetic Resonance
Imaging. Inorg. Chem..

[33] Thiele N. A., Woods J. J., Wilson J. J. (2019). Implementing f-Block Metal Ions in
Medicine: Tuning the Size Selectivity of Expanded Macrocycles. Inorg. Chem..

[34] Roca-Sabio A., Mato-Iglesias M., Esteban-Gómez D., Tóth É., Blas A. D., Platas-Iglesias C., Rodríguez-Blas T. (2009). Macrocyclic
Receptor Exhibiting Unprecedented Selectivity for Light Lanthanides. J. Am. Chem. Soc..

[35] Hu A., MacMillan S. N., Wilson J. J. (2020). Macrocyclic Ligands with an Unprecedented
Size-Selectivity Pattern for the Lanthanide Ions. J. Am. Chem. Soc..

[36] Aluicio-Sarduy E., Thiele N. A., Martin K. E., Vaughn B. A., Devaraj J., Olson A. P., Barnhart T. E., Wilson J. J., Boros E., Engle J. W. (2020). Establishing Radiolanthanum
Chemistry for Targeted
Nuclear Medicine Applications. Chem. - Eur.
J..

[37] Trommer J., Ullrich M., Reissig F., Brühlmann S. A., Nitt-Weber A.-K., Novy Z., Hajduova K., Kurfurstova D., Hendrychova R., Bouchal J. (2025). It’s
a match:
use of the radionuclide theranostic pair 133La/225Ac for the radiopharmacological
characterization of EGFR-targeted single-domain antibodies. EJNMMI Radiopharm. Chem..

[38] Aluicio-Sarduy E., Barnhart T. E., Weichert J., Hernandez R., Engle J. W. (2021). Cyclotron-Produced 132La as a PET
Imaging Surrogate
for Therapeutic 225Ac. J. Nucl. Med..

[39] Aluicio-Sarduy E., Hernandez R., Olson A. P., Barnhart T. E., Cai W., Ellison P. A., Engle J. W. (2019). Production and in vivo PET/CT imaging
of the theranostic pair 132/135La. Sci. Rep..

[40] Hu A., Martin K. E., Śmiłowicz D., Aluicio-Sarduy E., Cingoranelli S. J., Lapi S. E., Engle J. W., Boros E., Wilson J. J. (2023). Construction of the Bioconjugate
Py-Macrodipa-PSMA
and Its In Vivo Investigations with Large 132/135La3+ and Small 47Sc3+
Radiometal Ions. Eur. J. Inorg. Chem..

[41] Brühlmann S., Kreller M., Pietzsch H.-J., Kopka K., Mamat C., Walther M., Reissig F. (2022). Efficient Production of the PET Radionuclide
133La for Theranostic Purposes in Targeted Alpha Therapy Using the
134Ba­(p,2n)­133La Reaction. Pharmaceuticals.

[42] Nelson B.
J. B., Wilson J., Andersson J. D., Wuest F. (2020). High yield cyclotron
production of a novel 133/135La theranostic pair for nuclear medicine. Sci. Rep..

[43] Kurakina E. S., McNeil B. L., Khushvaktov J., Temerbulatova N. T., Mirzayev N. A., Magomedbekov E. P., Hoehr C., Ramogida C. F., Filosofov D. V., Radchenko V. (2025). Production and purification of radiolabeling-ready
132/135La from the irradiation of metallic natBa targets with low
energy protons. Nucl. Med. Biol..

[44] Shirpour A., Hadadi A., Zolghadri S., Vosoughi S., Rajabifar S. (2025). Preclinical
evaluation of [13xLa]­La-FAP-2286 as a novel theranostic agent for
tumors expressing fibroblast activation protein. Sci. Rep..

[45] Nelson B. J. B., Ferguson S., Wuest M., Wilson J., Duke M. J. M., Richter S., Soenke-Jans H., Andersson J. D., Juengling F., Wuest F. (2022). First In Vivo and Phantom
Imaging
of Cyclotron-Produced 133La as a Theranostic Radionuclide for 225Ac
and 135La. J. Nucl. Med..

[46] Lubberink M., Lundqvist H., Tolmachev V. (2002). Production, PET performance and dosimetric
considerations of^134^Ce/^134^La, an Auger electron
and positron-emitting generator for radionuclide therapy. Phys. Med. Biol..

[47] Baum R. P., Singh A., Benešová M., Vermeulen C., Gnesin S., Köster U., Johnston K., Müller D., Senftleben S., Kulkarni R. H. (2017). Clinical evaluation
of the radiolanthanide terbium-152: first-in-human PET/CT with 152
Tb-DOTATOC. Dalton Trans..

[48] Bailey T. A., Mocko V., Shield K. M., An D. D., Akin A. C., Birnbaum E. R., Brugh M., Cooley J. C., Engle J. W., Fassbender M. E. (2021). Developing the 134Ce and 134La pair as companion
positron emission tomography diagnostic isotopes for 225Ac and 227Th
radiotherapeutics. Nat. Chem..

