# Supplementary material for: Improved 140Nd Production for the 140Nd/140Pr In Vivo Generator through Target Recycling and Radiochemical Optimization
Source: ACS Omega. 2026 Jul 18;11(30):44998–5007. doi: 10.1021/acsomega.6c03114 (PMC13449204; doi:10.1021/acsomega.6c03114)
Supplement: Supplementary file 1 [file ao6c03114_si_001.pdf]

Improved ^{140}Nd Production for the ^{140}Nd/^{140}Pr In Vivo Generator Through Target Recycling and Radiochemical Optimization

Mangi Lal Godara[a,b], Gabriel Dufour[b], Anna Buryachenko[c], Suzanne E. Lapi[a,b]*

[a]Department of Chemistry, University of Alabama at Birmingham, AL, 35294

[b]Department of Radiology, University of Alabama at Birmingham, AL, 35294

[c]Department of Chemical and Materials Engineering, San Jose State University, CA, 95192

* Corresponding author: E-mail: lapi@uab.edu. Telephone: (205) 975-8689. Address: 1824 6th Ave. S., WTI 310F, Birmingham, AL 35294.

Supporting Information:

Table S1: Stable impurities in commercially sourced ^{141}Pr metal foils used for ^{140}Nd production analyzed by ICP-MS.

Element	Percentage (%) content in ^{141}Pr target	SD (%)
La	0.011	0.001
Ce	0.050	0.002
Nd	1.047	0.037
Sm	< 0.001	N.A.
Eu	< 0.001	N.A.
Gd	1.075	0.028
Tb	< 0.001	N.A.
Dy	< 0.001	N.A.
Ho	< 0.001	N.A.
Er	< 0.001	N.A.
Tm	< 0.001	N.A.
Yb	< 0.001	N.A.
Lu	< 0.001	N.A.
Cr	0.022	0.006
Fe	0.300	0.012
Co	0.014	0.002
Ni	0.019	0.000
Cu	0.187	0.008
Pb	0.625	0.002

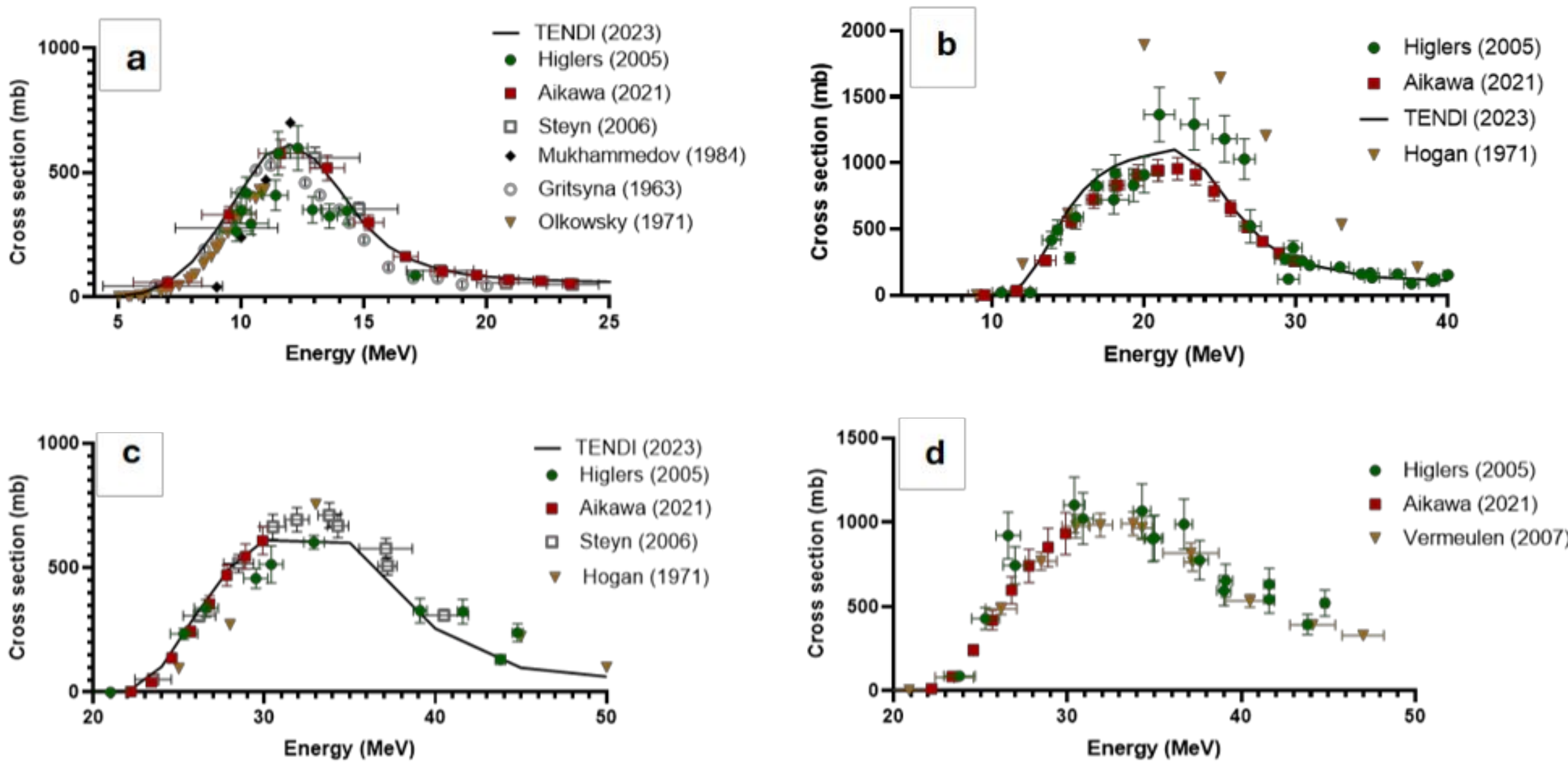

Figure S1: Cross-sections of various reaction routes accessible via proton irradiation of ^{141}Pr target material. **(a)** Cross-sections of ^{141}Pr(p,n)^{141}Nd. **(b)** Cross-sections of ^{141}Pr(p,2n)^{140}Nd. **(c)** Cross-sections of ^{141}Pr(p,3n)^{139m}Nd. **(d)** Cross-section of ^{141}Pr(p,x)^{139}Ce[1-8].

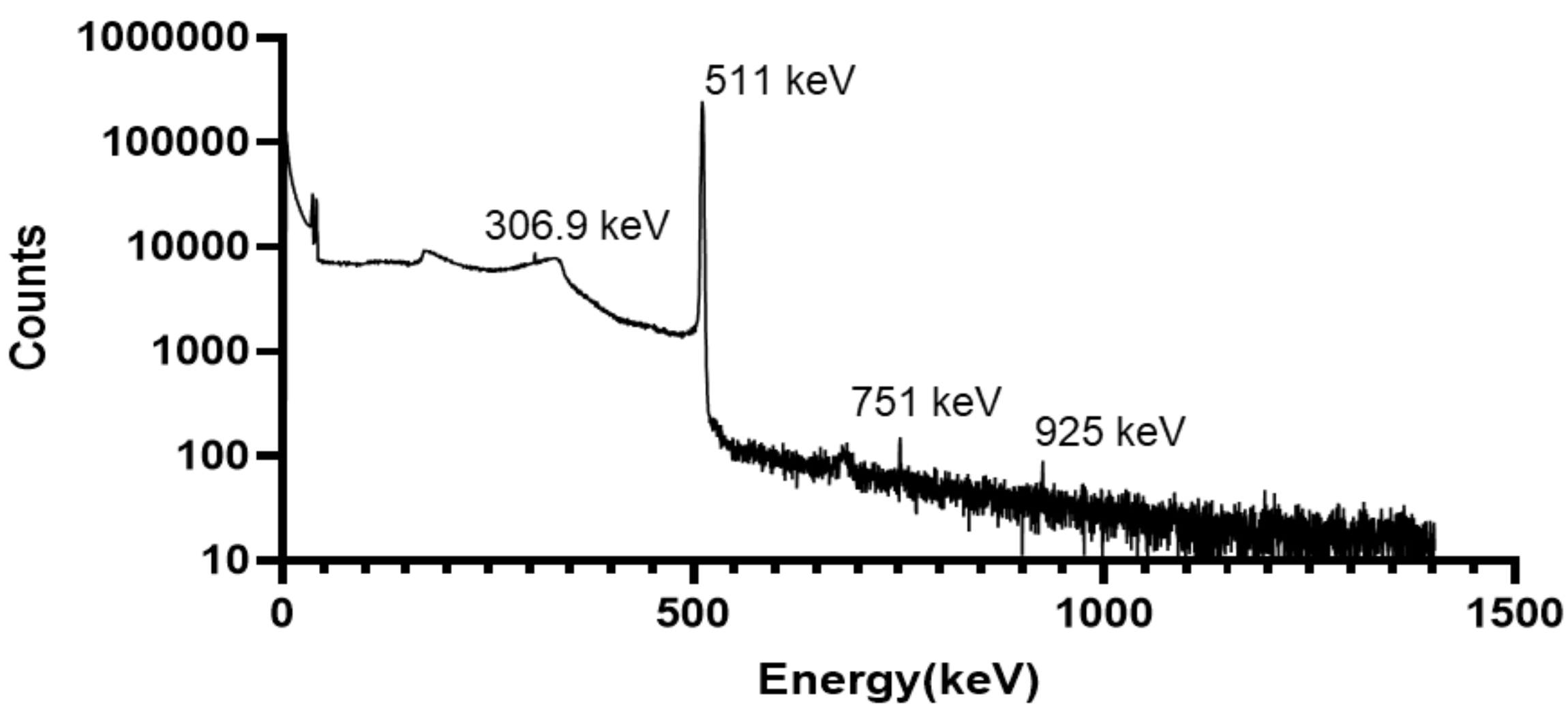

Figure S2: Gamma ray spectrum of aliquot taken from irradiated and digested Pr_6O_{11} pellet 48 h after the end of bombardment.

Weight Distribution (D_w) Studies:

Methods:

To identify optimal solvent conditions for neodymium and praseodymium separation using DGA normal resin, distribution coefficient (D_w) studies were performed across a range of hydrochloric acid based solvent systems. The investigated systems consisted of HCl alone; HCl combined with a constant concentration of NaCl (1 M); HCl combined with varying concentrations of NaCl; HCl combined with 10 % ethanol (EtOH); HCl combined with a constant concentration of 2-hydroxyisobutyric acid (HIBA, 0.2 M); HCl combined with a constant concentration of diethylenetriaminepentaacetic acid (DTPA, 0.2 M); HCl combined with a constant concentration of citrate (0.2 M); and HCl combined with 10 % hexafluoroisopropanol (HFIP). For each solvent condition, five tubes were prepared containing 2 mL of solvent per tube. Three tubes contained approximately 100 mg of DGA normal resin and 10 ppm each of Nd and Pr for experimental measurements. One tube containing resin only served as a blank, and one tube containing 10 ppm each of Nd and Pr without resin was used to determine the initial metal concentration. All tubes were vortexed, placed on a laboratory rocker, and equilibrated overnight. Following equilibration, samples were filtered through 0.2 µm nylon membrane filters, and aliquots were analyzed by ICP-MS to determine elemental concentrations. The weight distribution coefficient (D_w) for Nd and Pr was calculated using the equation:

$$Dw = \frac{Ci - Cf}{Cf} \times \frac{V}{W} \times 1000$$

Where Ci represents the initial metal concentration determined from tubes without resin, and Cf represents the final metal concentration averaged from the three resin-containing tubes. Concentrations were corrected by subtracting background values measured in the resin-only blank tube. In this equation, V is the solution volume in milliliters (2 mL), and W is the mass of resin in milligrams. Separation factors were calculated as the ratio of Dw for Nd to D_w for Pr for each solvent condition.

Results:

Weight distribution coefficient Nd and Pr in DGA normal resin across the various concentration of HCl was investigated and separation factors (D_wNd/D_wPr) were calculated to find high separation factor system. Across the investigated systems, separation factors were generally similar (Figure S2). The highest separation factors within each solvent group ranged between ~3.3 and 3.6, including 3.36 ± 0.03 for 3 M HCl, 3.36 ± 0.01 for 2.5 M HCl with 1 M NaCl, 3.47 ± 0.04 for 3 M HCl with 3 M NaCl, 3.33 ± 0.02 for 2.5 M HCl with 10 % ethanol, 3.42 ± 0.04 for 3 M HCl with 0.2 M HIBA, and 3.56 ± 0.04 for 3 M HCl with 0.2 M citrate. HCl solvent systems containing strong complexing agents such as DTPA (2.88 ± 0.01), and HFIP (2.23 ± 0.31) did not provide improved separation. Therefore, for simplicity and compatibility with subsequent column operations, HCl only systems were selected for further optimization. Based on the observed Dw

values, HCl concentrations between 2.5 M and 3 M were identified as suitable conditions for continued column separation optimization.

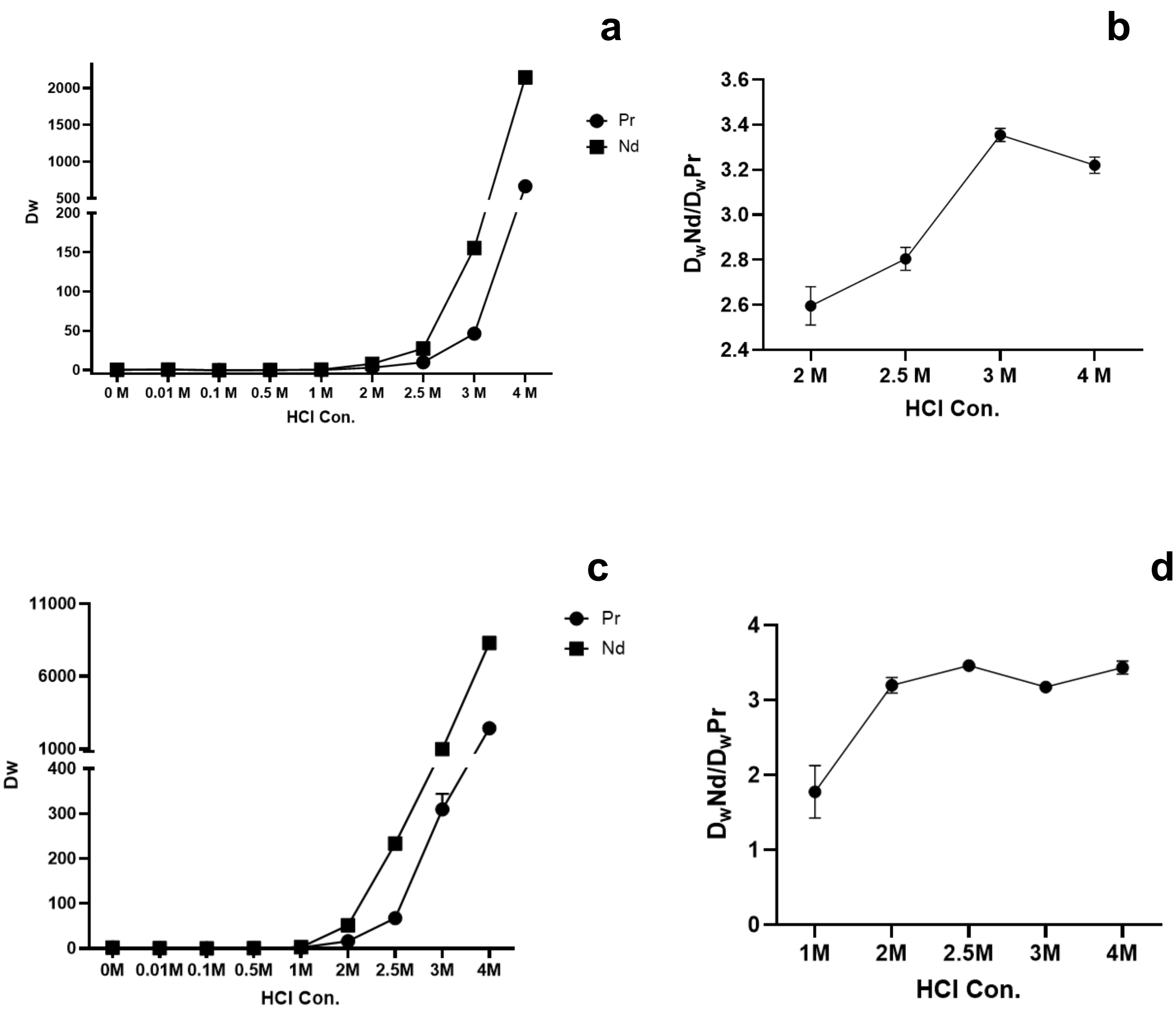

e

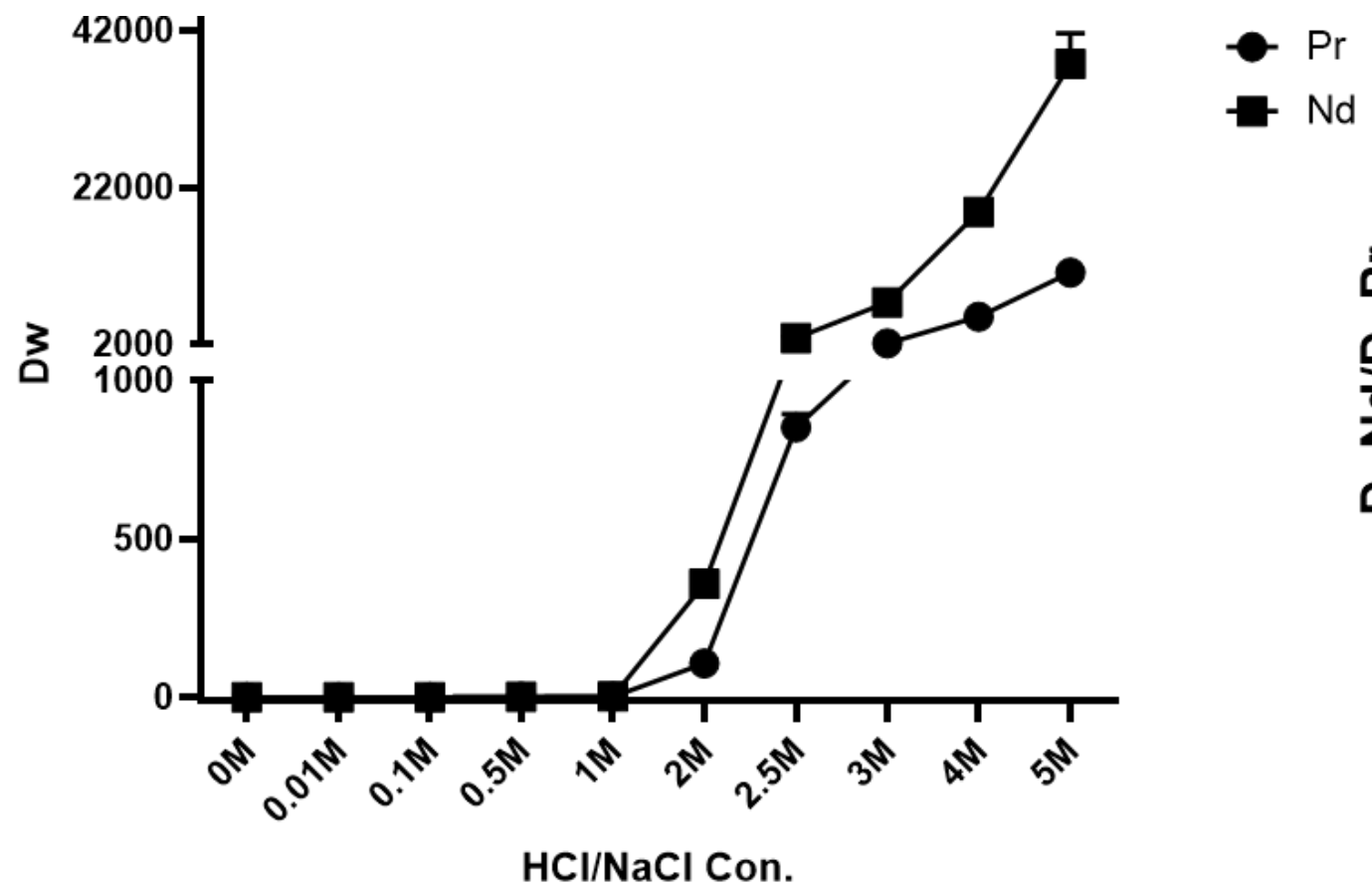

Pr
Nd
42000
22000
2000
1000
500
0
Dw
0M
0.01M
0.1M
0.5M
1M
2M
2.5M
3M
4M
5M
HCl/NaCl Con.

f

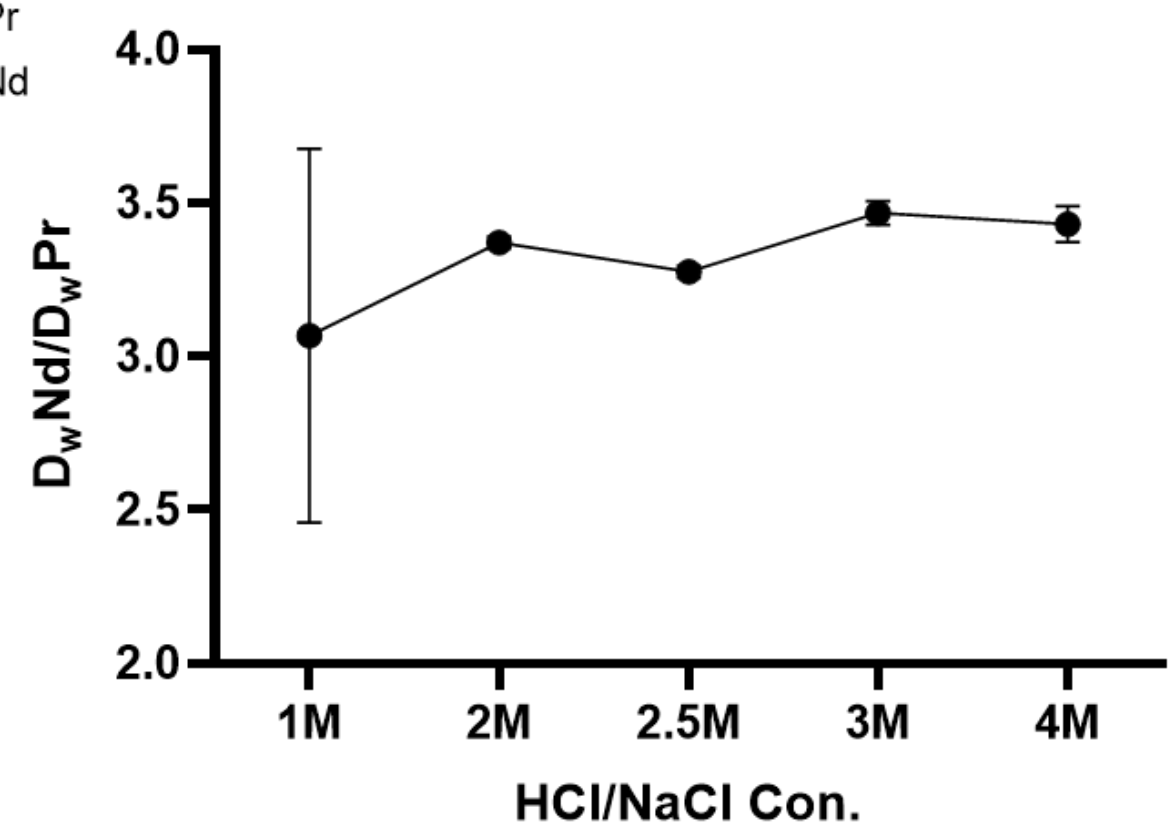

4.0
3.5
3.0
2.5
2.0
D_wNd/D_wPr
1M
2M
2.5M
3M
4M
HCl/NaCl Con.

g

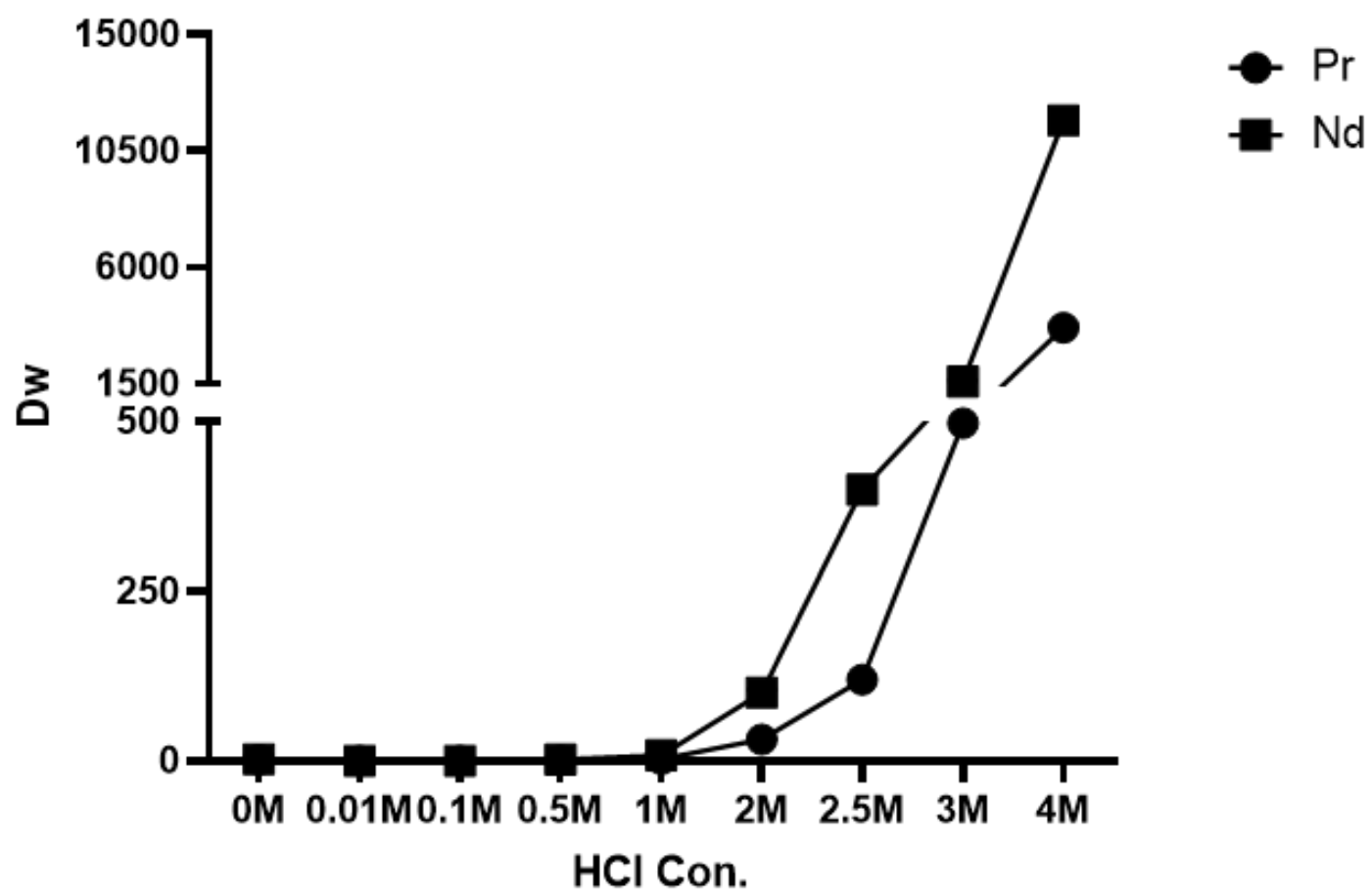

Pr
Nd
15000
10500
6000
1500
500
250
0
Dw
0M
0.01M
0.1M
0.5M
1M
2M
2.5M
3M
4M
HCl Con.

h

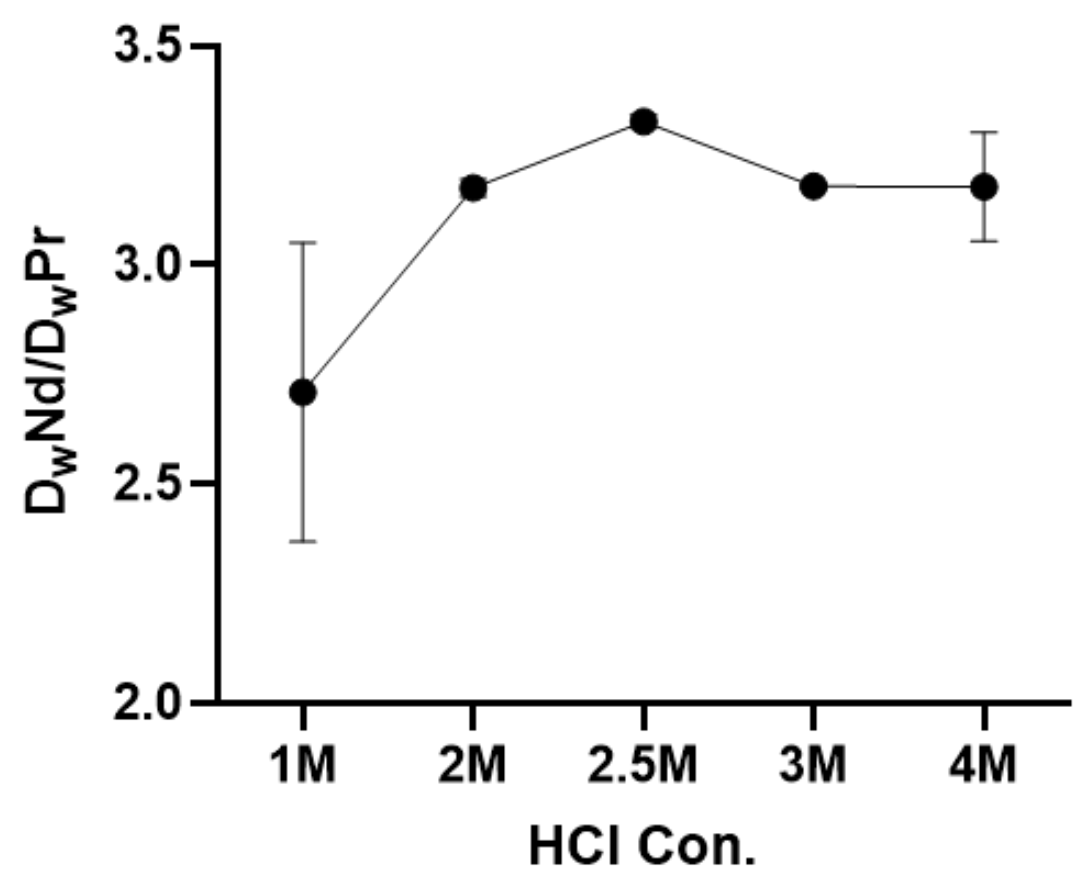

3.5
3.0
2.5
2.0
D_wNd/D_wPr
1M
2M
2.5M
3M
4M
HCl Con.

i

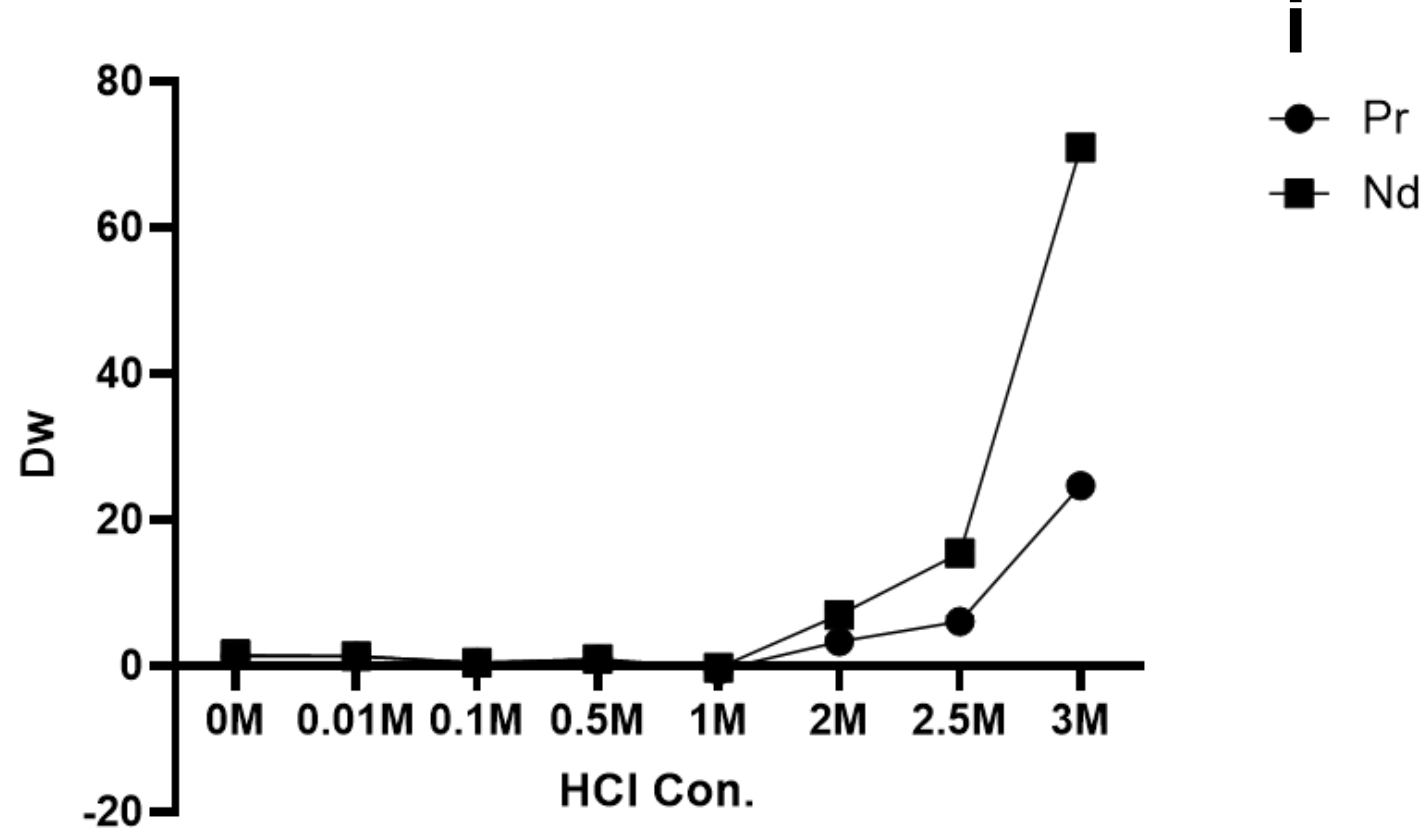

Pr
Nd
80
60
40
20
0
-20
Dw
0M
0.01M
0.1M
0.5M
1M
2M
2.5M
3M
HCl Con.

j

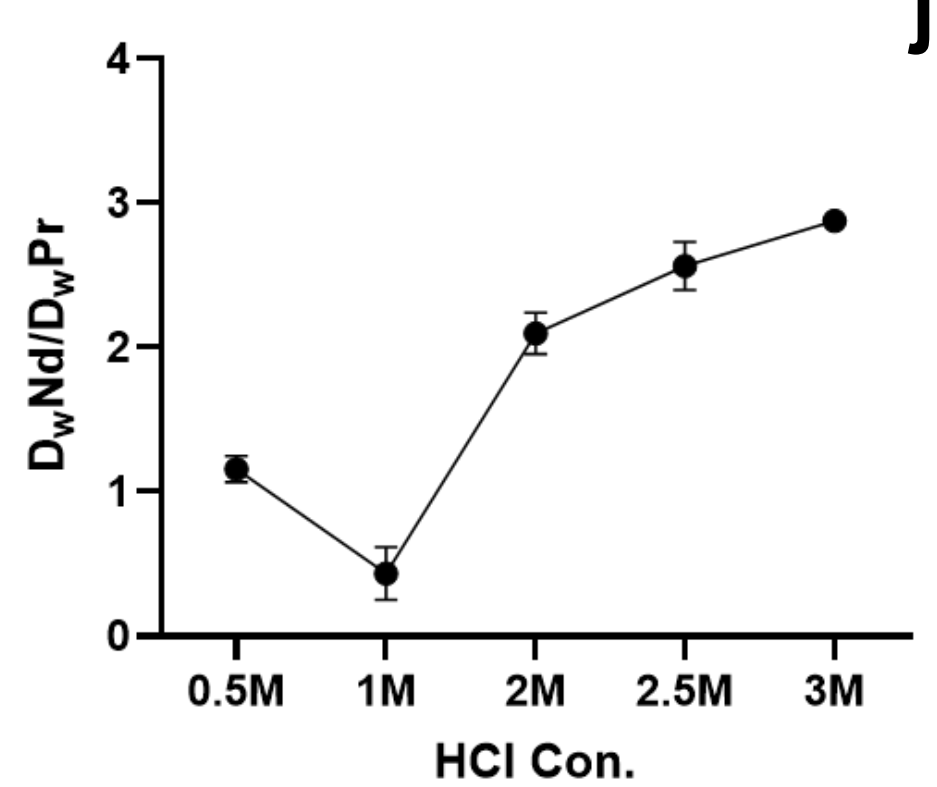

4
3
2
1
0
D_wNd/D_wPr
0.5M
1M
2M
2.5M
3M
HCl Con.

k

l

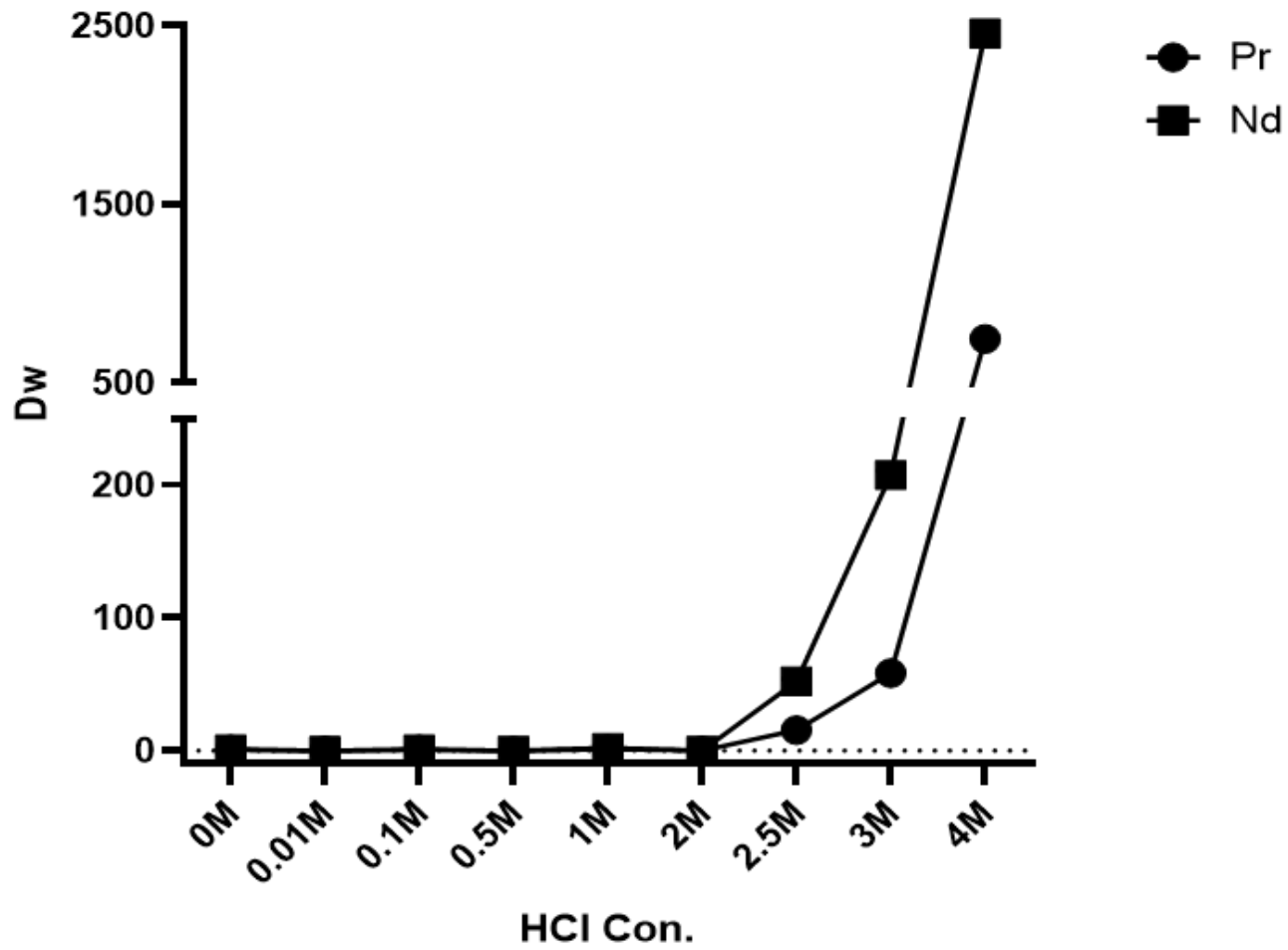
Dw
HCl Con.
Pr
Nd

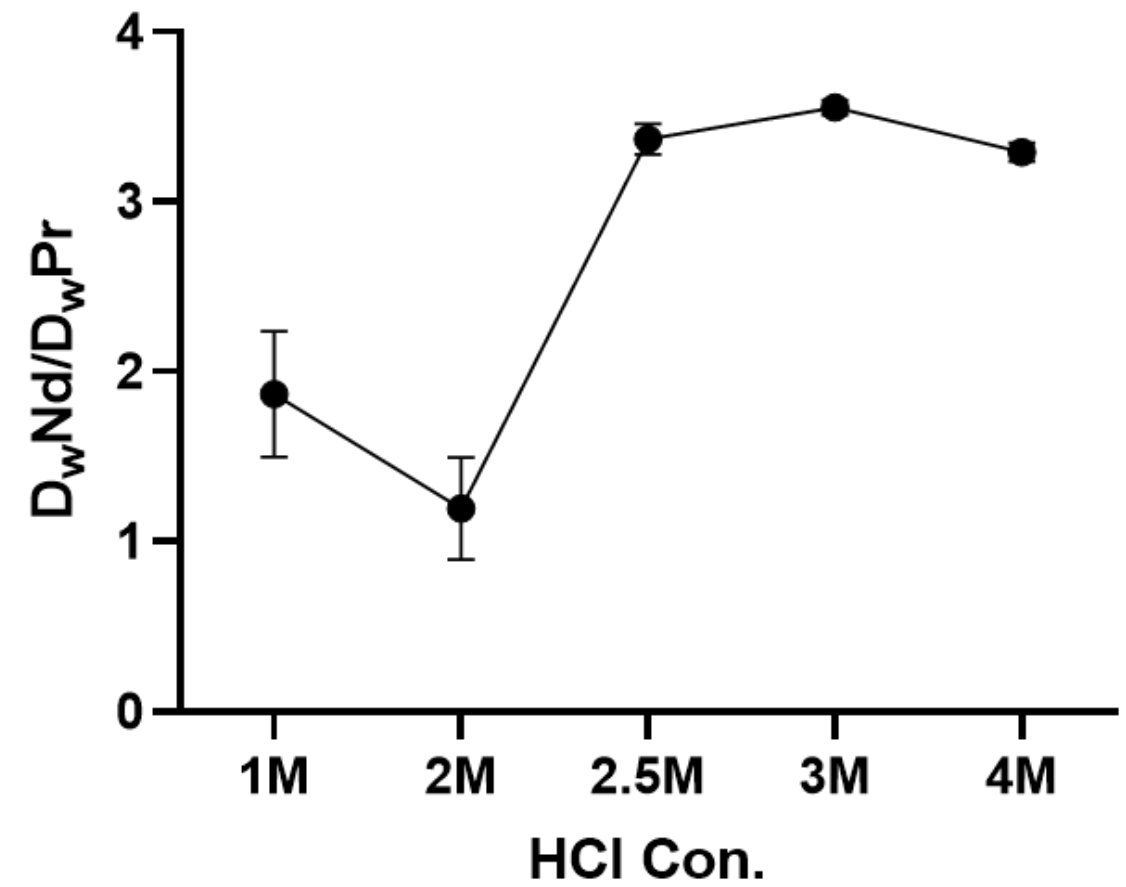
D_wNd/D_wPr
HCl Con.

m

n

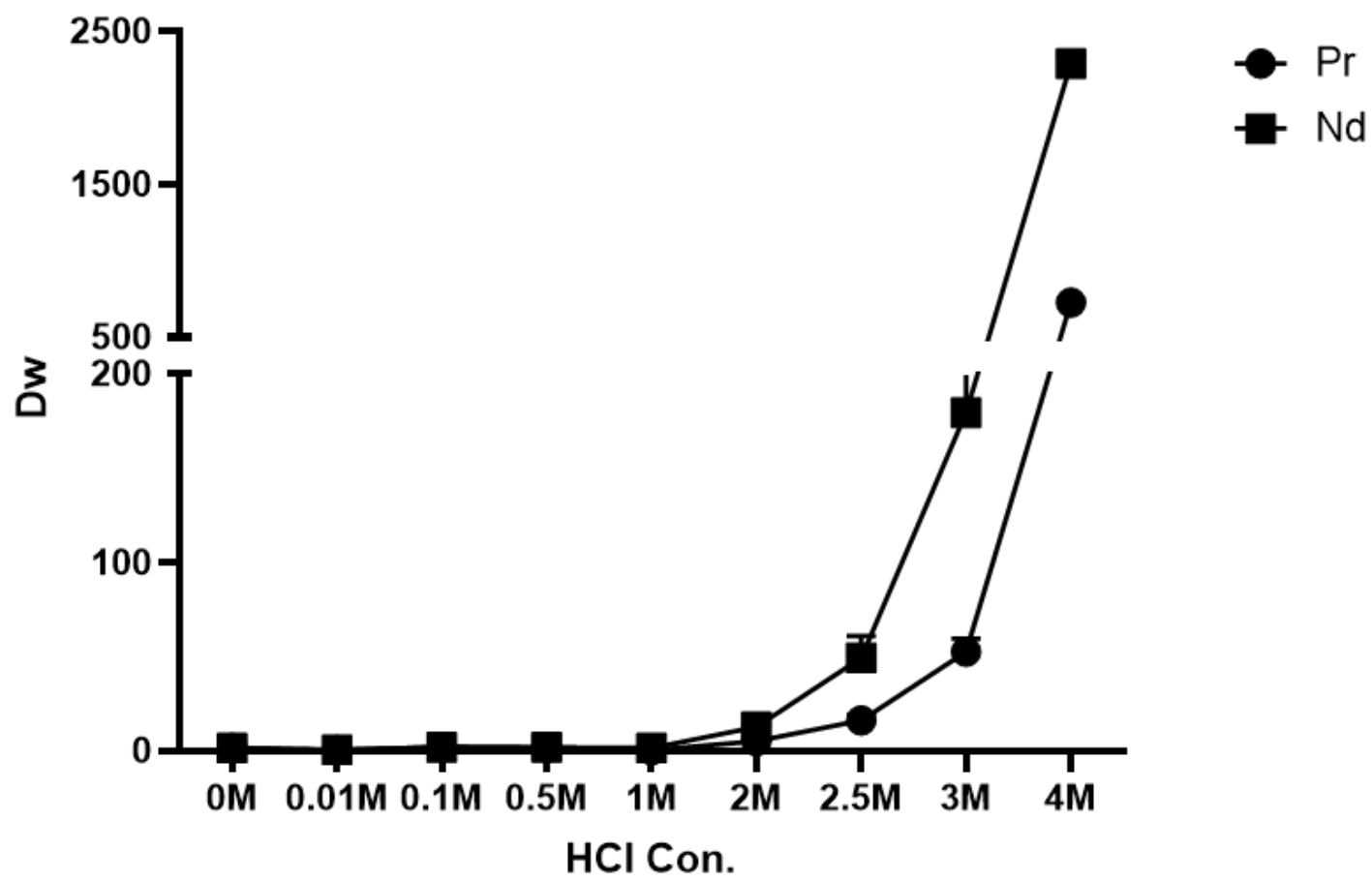
Dw
HCl Con.
Pr
Nd

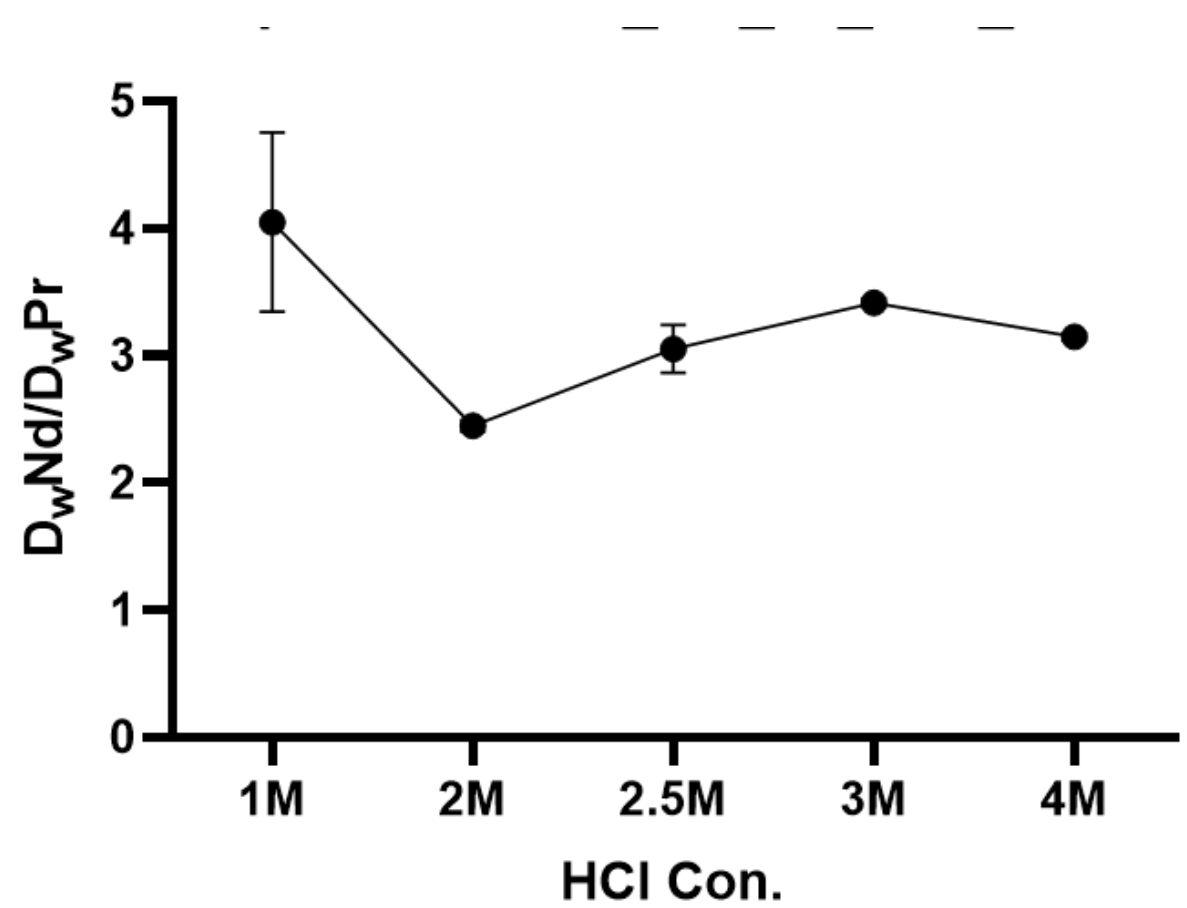
D_wNd/D_wPr
HCl Con.

o

p

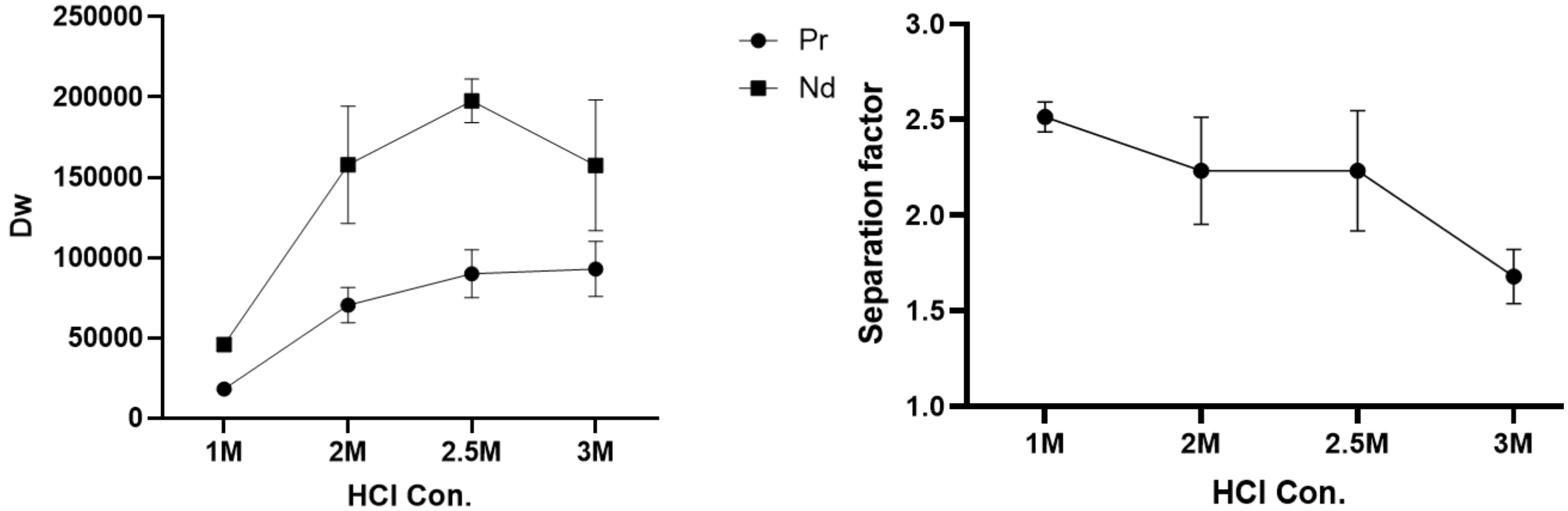

Figure S1: Weight distribution coefficients (D_w) of Nd and Pr and the resulting Nd/Pr separation factors on DGA normal resin in different HCl based solvent systems. (a) D_w values in HCl alone. (b) Corresponding separation factors in HCl alone. (c) D_w values in HCl with 1 M NaCl. (d) Corresponding separation factors in HCl with 1 M NaCl. (e) D_w values in HCl with varying concentrations of NaCl. (f) Corresponding separation factors in HCl with varying concentrations of NaCl. (g) D_w values in HCl with 10 % ethanol (EtOH). (h) Corresponding separation factors in HCl with 10 % EtOH. (i) D_w values in HCl with 0.2 M diethylenetriaminepentaacetic acid (DTPA). (j) Corresponding separation factors in HCl with 0.2 M DTPA. (k) D_w values in HCl with 0.2 M citrate. (l) Corresponding separation factors in HCl with 0.2 M citrate. (m) D_w values in HCl with 0.2 M 2-hydroxyisobutyric acid (HIBA). (n) Corresponding separation factors in HCl with 0.2 M HIBA. (o) D_w values in HCl with 10% hexafluoroisopropanol (HFIP). (p) Corresponding separation factors in HCl with 10 % HFIP.

1. Aikawa, M., Hanada, Y., Huang, H., et al., *Activation cross sections of proton-induced reactions on praseodymium up to 30 MeV.* Nuclear Instruments and Methods in Physics Research Section B: Beam Interactions with Materials and Atoms, 2021; **508**29–33.
2. Hilgers, K., Shubin, Y.N., Coenen, H.H., et al., *Experimental measurements and nuclear model calculations on the excitation functions of natCe(3He, xn) and 141Pr(p, xn) reactions with special reference to production of the therapeutic radionuclide 140Nd.* Radiochimica Acta, 2005–09–01; **93**(9-10).
3. Hogan, J.J., *Study of the 141Pr(p,xn) reaction from 10–85 MeV.* Journal of Inorganic and Nuclear Chemistry, 1971/11/01; **33**(11).
4. Koning, A.J., Rochman, D., Sublet, J.-C., et al., *TENDL: Complete Nuclear Data Library for Innovative Nuclear Science and Technology.* Nuclear Data Sheets, 2019/01/01; **155**.
5. Mukhammedov, S., Vasidov, A., Pardaev, É., et al., *Use of proton and deuteron activation methods of analysis in the determination of elements with Z>42.* Soviet Atomic Energy 1984 56:1, 1984/01; **56**(1).
6. Olkowsky, J., Et, M.L.P.,Gratot, I., *Fonction d'excitation de 141Pr (p, n) 141Nd Jusqu'à 11.1 MeV.* Nuclear Physics, 1961/02/01; **23**.

7. Steyn, G.F., Vermeulen, C., Nortier, F.M., et al., *Production of no-carrier-added 139Pr via precursor decay in the proton bombardment of natPr.* Nuclear Instruments and Methods in Physics Research Section B: Beam Interactions with Materials and Atoms, 2006/11/01; **252**(2).
8. Vermeulen, C., Steyn, G.F., Nortier, F.M., et al., *Production of 139Ce by proton-induced reactions on 141Pr and natLa.* Nuclear Instruments and Methods in Physics Research Section B: Beam Interactions with Materials and Atoms, 2007/02/01; **255**(2).
